# HDAC Inhibition Induces CD26 Expression on Multiple Myeloma Cells via the c-Myc/Sp1-mediated Promoter Activation

**DOI:** 10.1158/2767-9764.CRC-23-0215

**Published:** 2024-02-09

**Authors:** Hiroko Nishida, Reiko Suzuki, Kiyora Nakajima, Mutsumi Hayashi, Chikao Morimoto, Taketo Yamada

**Affiliations:** 1 Department of Pathology, Keio University School of Medicine, Shinjuku-ku, Tokyo, Japan.; 2 Division of Hematology, Department of Internal of Medicine, Keio University School of Medicine, Shinjuku-ku, Tokyo, Japan.; 3 Department of Collaborative Research Resources, Keio University School of Medicine, Shinjuku-ku, Tokyo, Japan.; 4 Department of Therapy Development and Innovation for Immune Disorders and Cancers, Juntendo University, Graduate School of Medicine, Bunkyo-ku, Tokyo, Japan.; 5 Department of Pathology, Faculty of Medicine, Saitama Medical University, Saitama, Japan.

## Abstract

CD26 is ubiquitously and intensely expressed in osteoclasts in patients with multiple myeloma, whereas its expression in plasma cells of patients with multiple myeloma is heterogeneous because of its cellular diversity, immune escape, and disease progression. Decreased expression levels of CD26 in myeloma cells constitute one of the mechanisms underlying resistance to humanized anti-CD26 mAb therapy in multiple myeloma. In the current study, we show that histone deacetylase inhibition (HDACi) with broad or class-specific inhibitors involves the induction of CD26 expression on CD26^neg^ myeloma cells both transcriptionally and translationally. Furthermore, dipeptidyl peptidase Ⅳ (DPPⅣ) enzymatic activity was concomitantly enhanced in myeloma cells. Combined treatment with HDACi plus CD26mAb synergistically facilitated lysis of CD26^neg^ myeloma cells not only by antibody-dependent cellular cytotoxicity but also by the direct effects of mAb. Of note, its combination readily augmented lysis of CD26^neg^ cell populations, refractory to CD26mAb or HDACi alone. Chromatin immunoprecipitation assay revealed that HDACi increased acetylation of histone 3 lysine 27 at the CD26 promoter of myeloma cells. Moreover, in the absence of HDACi, c-Myc was attached to the CD26 promoter via Sp1 on the proximal G-C box of myeloma cells, whereas, in the presence of HDACi, c-Myc was detached from Sp1 with increased acetylation of c-Myc on the promoter, leading to activation of the CD26 promoter and initiation of transcription in myeloma cells. Collectively, these results confirm that HDACi plays crucial roles not only through its anti-myeloma activity but by sensitizing CD26^neg^ myeloma cells to CD26mAb via c-Myc/Sp1-mediated CD26 induction, thereby augmenting its cytotoxicity.

**Significance:**

There is a desire to induce and sustain CD26 expression on multiple myeloma cells to elicit superior anti-myeloma response by humanized anti-CD26 mAb therapy. HDACi upregulates the expression levels of CD26 on myeloma cells via the increased acetylation of c-MycK323 on the CD26 promoter, leading to initiation of CD26 transcription, thereby synergistically augments the efficacy of CD26mAb against CD26^neg^ myeloma cells.

## Introduction

The therapeutic landscape for multiple myeloma has dramatically changed over the past decades and recent progress in the treatment options for multiple myeloma, in particular with the incorporation of anti-CD38 targeting mAbs into standard care regimens including proteasome inhibitors (PI) and immunomodulatory drugs (IMiD) has tremendously improved the prognosis of patients with multiple myeloma (1–3). However, the vast majority of patients still relapse and become refractory to existing treatments due to the heterogeneity of multiple myeloma, in which multiple clones have different clinical behaviors as well as acquired resistance (4). Notably, triple class–exposed patients with multiple myeloma typically have progressively shorter durations of responses (DOR) with subsequent lines of therapy and penta-class-refractory patients have extremely dismal outcomes with a median progression-free survival of 3 months and overall survival (OS) of less than 6 months (4). Therefore, the treatment of patients with relapsed or refractory multiple myeloma (RRMM) remains a major challenge and highlights the urgent need for the development of novel effective treatments to target alternative antigens or treatments with different mechanisms of action.

In recent years, in addition to naked mAbs, several novel targeted immunotherapies, including antibody–drug conjugates (ADC), bispecific antibodies (BsAb), and chimeric antigen receptor T-cells (CAR-T), have been developed to eliminate myeloma cells (5–7). B-cell maturation antigen, BCMA/TNFRSF17 is highly expressed on most of malignant plasma cells and represents a promising novel target for multiple myeloma therapy (8). To date, three BCMA-directed therapies have been approved for patients with RRMM who have undergone at least four prior lines of therapy including PIs, IMiDs, and anti-CD38 mAbs (9–18). Belentamab mafodotin (belamaf), a first-in-class humanized, afucosylated IgG_1_ BCMA-targeted ADC containing monomethyl auristatin F, eliminates myeloma cells by a multi-modal mechanism of action via direct myeloma cell killing and an anti-myeloma immune response (9). Belamaf has shown promising efficacy as a single agent, with an overall response rate of 32% and a DOR of 12.5 months in triple-refractory patients with RRMM (10–12). However, its clinical use is limited because of suboptimal disease control and the high incidence of off-target adverse events such as ocular toxicities and pancytopenia (5, 8–12).

Moreover, the substantial efficacy of cell-based immunotherapies that engage T cells to BCMA-expressing myeloma cells and redirect subsequent lysis of myeloma cells have recently been uncovered by the emergence of BCMA-directed CAR-T cell and BsAb constructs (8, 13, 14). Two BCMA-directed CAR-T cell therapies, idecabtagene vicleucel (ide-cel, abecma) and ciltacabtagene-autoleucel (cilta-cel) have shown remarkable efficacy with rapid, deep, and durable clinical responses. Similarly, a humanized BsAbs with dual binding sites targeting both CD3 expressed on T cells and BCMA on myeloma cells, Teclistamab (cqyv, JNJ64007957) has proven highly active as a single agent with a deep and durable response. All of these immunotherapies result in a substantial improvement of outcomes in triple class–exposed patients with RRMM (14–18).

CD26, a 110-kDa transmembrane glycoprotein with dipeptidyl peptidase Ⅳ (DPPⅣ) activity (19–22), is expressed on several tumor cells, including malignant lymphoma, and has been implicated in T-cell activation and tumorigenesis (23, 24). In first-in human phase I study, recombinant humanized anti-CD26mAb was generally well tolerated and revealed antitumor effects without significant side effects in 33 patients with advanced CD26-expressing tumors, including renal cell carcinoma (*n* = 9), malignant pleural mesothelioma (MPM, *n* = 23), and urothelial carcinoma (*n* = 1; ref. 25). Furthermore, CD26mAb also revealed modest antitumor efficacy, with partial remission in 1 patient and stable disease (SD) in 14 patients, leading to a median OS of 9.7 months in 31 Japanese patients with advanced MPM (26, 27). On the other hand, the roles of CD26 in plasma cell malignancies remain elusive. Recently, we identified that CD26 is uniformly and intensely expressed in osteoclasts, whereas its expression in the plasma cells of patients with multiple myeloma was heterogeneous, leading to marked differences of response to CD26mAb therapy in multiple myeloma (28, 29). Decreased expression levels of CD26 in myeloma cells is one of the mechanisms underlying innate or acquired resistance to CD26mAb therapy in multiple myeloma. Therefore, more detailed understanding of both host- and tumor-related factors that predict the response to this mAb may result in the novel design of CD26-based immunotherapeutic approach for boosting cytotoxic efficacy in RRMM.

Histone deacetylases (HDAC) are highly expressed in various cancer cells and regulate aberrant gene transcription, which contributes to tumorigenesis. Therefore, HDAC inhibition (HDACi) can restore the gene transcription, that is aberrantly expressed in cancer cells, leading to cell cycle arrest, cell differentiation, and apoptosis (30, 31). Moreover, HDACi also epigenetically modifies the expression of cell surface or immunomodulatory molecules in various cancer cells and immune effector cells (30–35).

In the current study, we elucidated for the first time the potential impacts and mechanisms of HDACi by isoform-selective as well as broad inhibitors on the regulation of CD26 expression in myeloma cells, thereby eliciting superior anti-myeloma efficacy by CD26mAb. We demonstrated that HDACi mediates c-Myc acetylation on the CD26 promoter of myeloma cells, which leads to activation of the promoter and initiation of CD26 transcription in myeloma cells as one of mechanisms for the induction of CD26 in myeloma cells. Our results point to a novel observation on the role of HDACi and highlight that the combination of an isoform-selective HDACi plus CD26mAb confers attractive therapeutic strategies by resensitizing CD26^neg^ myeloma cells to CD26mAb and augmenting its cytotoxic efficacy, thereby overcoming therapeutic resistance to mAb in RRMM.

## Materials and Methods

### Cell Lines

Five multiple myeloma cell lines: KMS26, 27, 28, and RPMI8226 were obtained from the National Institute of Biomedical Innovation, Health and Nutrition (NIBIOHN, Osaka, Japan). KMS11 was obtained from ATCC. All cell lines were maintained in RPMI1640 (Invitrogen), containing 10% FBS (Life Technologies), 100 µg/mL penicillin, and 100 µg/mL streptomycin (Life Technologies) at 37°C in a humidified atmosphere of 5% CO_2_.Contamination of *Mycoplasma* was regularly examined by PCR, and no contamination was detected during experiments concerning this work.

### Reagents and Cells

Human bone marrow (BM) mononuclear cells (MNC) and peripheral blood mononuclear cells were purchased from Lonza and human natural killer (NK) cells were obtained from Biotherapy Institute of Japan (Tokyo, Japan). HDAC inhibitors; pan HDACi: panobinostat_50 µmol/L, vorinostat_1.0 µmol/L, isoform-selective HDACi: romidepsin (HDAC1i)_0.125 µmol/L, BG45 (HDAC1, 3i) 1.0 µmol/L, entinostat (HDAC1, 3i)_50 µmol/L, RG2833 (HDAC1, 3i)_0.5 µmol/L, nexturastat A (HDAC6i)_0.125 µmol/L, tubastatin A (HDAC6i)_25 µmol/L, ricolinostat (HDAC1, 3, 6i)_0.5 µmol/L were purchased from Selleck Chemical Co. LTD. for use as therapeutic agents. These compounds, reconstituted in DMSO were added to the medium in which myeloma cell lines were cultured at the indicated concentrations for indicated times from 24 to 72 hours. The CD26mAb, humanized IgG_1_, employed in the current study was generously provided by Y's AC. The CD26mAb was generated by utilizing the complementarity-determining regions of the murine anti-human CD26mAb, 14D10 with no cross-reactivity to murine CD26. Isotype IgG_1_ (Sigma-Aldrich) was used as a control. In the experiments, after the incubation with the treatment of each HDACi for 48 hours, CD26mAb was subsequently added to the medium in which myeloma cell lines were cultured at 10 µg/mL for 24 hours.

### Cell Viability Assay

Myeloma cell lines were treated with one of nine HDACi or isotype (iso) control IgG_1_ (BioLegend) and incubated for 48 hours, followed by the additional incubation with the treatment by isotype (iso) control IgG_1_ or CD26mAb at 10 µg/mL for 24 hours at 37°C in a humidified atmosphere of 5% CO_2_. At the end of each timepoints, myeloma cells were collected and cell viability was determined via the conversion of a soluble MTT [3-(4,5-dimethtlthiazol-2-yl)-2,5-diphenyltetrazolium bromide] to insoluble formazan using CellQuanti-MTT cell viability assay kit (BioAsssay Systems), according to the manufacturer's instructions. The absorbance of each well was measured at 560 nm with GloMax-Muluti Detection System (Promega).

### Apoptosis Assay

Apoptosis of myeloma cells was determined by staining cells with annexin and propidium iodide using Annexin V-FITC apoptosis detection kit (BioVision), according to the manufacturer's instructions. The intensity of each cell was analyzed by flow cytometry; CytoFLEX (Beckman Coulter).

### Antibody-dependent Cellular Cytotoxicity Assay

Five myeloma cell lines: KMS11, 26, 27, 28, and RPMI8226 (1 × 10^6^/mL), transduced with luciferase (Promega) were treated with one of nine HDACi at the indicated concentration iso control IgG_1_and incubated at 37°C for 48 hours. Subsequently, these cells were additionally incubated with iso control IgG_1_or CD26mAb (10 µg/mL) in the presence or absence of human NK effector cell at an effector to target (E/T) ratio of 20, at 37°C for 24 hours. Thereafter, d-luciferin substrate was added at 150 µg/mL and the bioluminescence (luciferase^+^ cells) was measured using GloMax-Muluti Detection System (Promega). The cell viability (%) was calculated as mean signal in the presence of CD26mAb plus effector NK cells with or without the treatment of each HDACi × 100/optical density (OD) signal in the control IgG_1_ and effector NK cells.

### Complement-dependent Cellular Assay

Myeloma cell lines were incubated with the treatment by each HDACi at the indicated concentration or iso control IgG_1_ for 48 hours, followed by the additional incubation with the treatment by either CD26mAb (10 µg/mL) or iso control IgG_1_ in the presence or absence of 50% fresh human serum as a source of complement at 37°C for 1 hour. Cell viability was measured by MTT assay using CellQuanti-MTT cell viability assay kit (BioAsssay systems), according to the manufacturer's instructions. The absorbance of each well was measured at 560 nm with GloMax-Muluti Detection System (Promega).

### Whole Transcriptome Profiling

The GeneChip Whole Transcript (WT) Pico Reagent Kit (Thermo Fisher Scientific) was used to prepare hybridization-ready targets of total RNA samples with GeneChip WT Expression Arrays (Thermo Fisher Scientific) according the user guide. Briefly, the assay workflow consists of three steps. First, after first-strand cDNA sysnthesis, 3′ adaptor cDNA synthesis, double-stranded (ds) cDNA sysnthesis and cRNA amplification by *in vitro* transcription of ds cDNA using T7 RNA polymerase, cRNA purification and quantification was performed. Subsequently, second cycle single-strand (ss-cDNA) synthesis and cRNA hydrolyzation by RNaseH were conducted followed by ss-cDNA purification and quantification, fragmentation and terminal labeling of ss-cDNA. Finally, hybridization to WT array was performed using GeneChip cartridge array according the user guide. Microarray signals were processed using a standard robust multi-array averaging algorithm. Observed signals were normalized using quantile normalization methods and genes that had no significant signals were ignored to reduce the signals.

### Chromatin Immunoprecipitation (ChIP)-qPCR

For chromatin immunoprecipitation (ChIP) assays, anti-histone H3, GTX122148 (GENETEX), anti-acetyl histone H3 (H3K27ac), #39134, 39336 (Active Motief), anti-Sp1, GTX110593 (GENETEX), A19649 (ABclonal), anti-c-Myc, A19032 (ABclonal), C15410174 (Diagenode), anti-c-MycK323ac, C15410346 (Diagenode) were used. The detailed procedures were provided in Supplementary Data S1 and Supplementary Table S1. qPCR analysis was performed using the Thermal Cycle Dice (Takara Bio). Several amplifications were performed by classic PCR and products were run on 1.5% agarose gels and was visualized on iBrightFL1000 (Thermo Fisher Scientific). Primer sequences were available in Supplementary Table S1.

### Statistical Analysis

All statistical analyses were performed using Student *t* test for two group comparisons and *P* values less than 0.05 were considered statistically significant. The data are presented as the mean values with 95% confidence intervals, and the results are representative of three independent experiments.

Supplementary Data S1 include details of protocols for immunophenotyping (Supplementary Table S2), quantitation and qualification of mRNA levels, IHC, immunoblotting, ELISA, and ChIP-qPCR.

### Data Availability

All data are available in the main text or Supplementary Data. Further information in this article is available from the corresponding authors on request.

## Results

### HDACi Increases the Expression of CD26 on Myeloma Cell Lines

**FIGURE 1:**
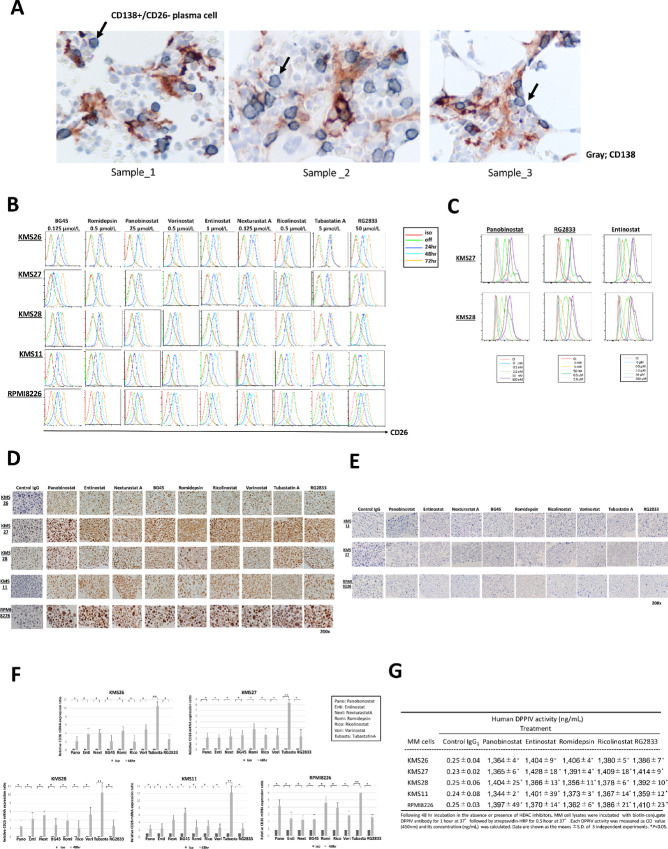
Induction of CD26 expression on myeloma cell lines by HDACi. **A,** CD26 expression in plasma cells of bone marrow tissues of patients with multiple myeloma. Analysis of primary BM samples from several patients with multiple myeloma revealed that CD138^pos^ plasma cells were rarely stained with CD26 (gray, CD138; red, CD26; original magnification, × 200). **B,** Flow cytometry with anti-CD26 (rat clone)-fluorescein (FITC) or isotype control IgG_1_ was performed in five myeloma cell lines KMS11, 26, 27, 28, and RPMI8226. Overlay histograms show CD26 expression on myeloma cell lines before and after treatment with one of nine HDACi for the indicated times (24, 48, 72, and 48 hours after subsequent removal of each HDACi) at the indicated doses. HDACi elicited exposure time-dependent upregulation of CD26 expression on myeloma cells, whereas subsequent removal of HDACi resulted in a decline of CD26 expression to the decreased or near-pretreatment levels. **C,** KMS27 and KMS28 was incubated with titrated concentrations of panobinostat (0.5, 5.0, 50, and 100 nmol/L), RG2833 (5, 50 nmol/L, 0.5, and 5.0 µmol/L) and entinostat (0.5, 5.0, 50, and 500 µmol/L) for 48 hours, after which cells were harvested to analyze the levels of surface CD26 expression in myeloma cells by flow cytometry. Overlay histogram shows CD26 expression on each myeloma cell before and after 48 hours of treatment with each HDACi at the indicated doses. HDACi elicited a dose-dependent upregulation of CD26 expression on myeloma cells, whereas 5.0 µmol/L of RG2833 and 500 µmol/L of entinostat did not further/significantly enhance CD26 expression on myeloma cells, compared with 0.5 µmol/L of RG2833 and 50 µmol/L of entinostat. **D,** Myeloma cell lines KMS11, 26, 27, 28, and RPMI8226 were immunohistochemically stained for CD26 before and after treatment with one of nine HDACi. All tested myeloma cell lines cultured alone without each HDACi were either slightly stained for CD26 or completely lacked CD26 expression. In contrast, cell lines treated with each HDACi for 48 hours revealed moderate to intense CD26 expression (CD26, brown stain; original magnification, × 200). **E,** Thereafter, removal of the HDACi for 48 hours resulted in CD26 expression to the decreased or near-pretreatment levels again (CD26; brown-stained; original magnification, × 200). **F,** Expression levels of CD26 mRNA in myeloma cell lines KMS11, 26, 27, 28, and RPMI8226 before and after the treatment of one of nine HDACi for 48 hours were analyzed using real-time quantitative RT-PCR assay with specific primers for CD26 (Supplementary Table S1). The CD26 mRNA transcription levels in myeloma cell lines treated with each HDACi revealed a significant increase, compared with those of untreated myeloma cells. Results are shown as ratio of CD26mRNA/GAPDH mRNA. Bar diagrams represent the mean values ± SE. *n* = 3; *, *P* < 0.05; **, *P* < 0.01. **G,** The levels of DPPⅣ activity in supernatants derived from myeloma cell lines KMS11, 27, 28, and RPMI8226, cultured in the presence or absence of one of nine HDACi for 48 hours were determined by ELISA. The DPPⅣ levels in supernatants of myeloma cells, which were incubated in the presence of each HDACi were significantly elevated, compared with those of control IgG_1_. The data represent the mean ± SE of triplicate wells from the representative of three independent experiments. The error bars represent the range, *, *P* < 0.05.

We have already shown that the BM tissues of patients with multiple myeloma contained intensely CD26-stained osteoclasts, whereas CD26 expression on plasma cells was heterogeneously distributed (28, 29). Indeed, analysis of primary BM tissues from multiple myeloma patient revealed that several CD138^pos^ plasma cells were stained with CD26, whereas other plasma cells were not (29). Moreover, those from several patients with multiple myeloma showed that CD138^pos^ plasma cells were rarely stained with CD26 (Fig. 1A). Therefore, it is not necessarily reasonable to target CD26 on myeloma cells by CD26-targeted immunotherapy to elicit extensive cytotoxicity against multiple myeloma. HDACi has the ability to modulate the expression of cell surface molecules such as tumor antigens or immunomodulatory molecules in tumor cells or immune effector cells (30–35). Consequently, we evaluated the effects of HDACi by broad or isoform-selective inhibitors on cell surface CD26 expression levels on myeloma cells. First, five myeloma cell lines KMS11, 26, 27, 28, and RPMI8226, were cultured in the presence or absence of HDACi; the broad inhibitors, panobinostat and vorinostat or the isoform-specific inhibitors, romidepsin (HDAC1i); BG45, entinostat and RG2833 (HDAC3i); and nexturastatA, ricolinostat, and tubastatinA (HDAC6i) for the indicated times (24, 48, 72 hours) and then, the expression levels of CD26 on myeloma cells were analyzed at each timepoints by flow cytometry. Although, cell surface CD26 expression levels on myeloma cell lines were relatively low or absent before treatment with each HDACi, an increase in CD26 expression levels was observed within 24 hours of the initiation of treatment. Moreover, CD26 levels increased further while exposure to each HDACi continued, and the maximum increase in CD26 expression was observed at 48 to 72 hours (Fig. 1B). Intriguingly, subsequent removal of the HDACi for 48 hours resulted in a decline of CD26 expression levels on myeloma cells to levels slightly positive or similar to pretreatment levels (Fig. 1B).

We also treated KMS27 and KMS28 with titrated concentrations of with panobinostat (0.5, 5.0, 50, 500 nmol/L), RG2833 (5.0, 50 nmol/L, 0.5, 1.0 µmol/L), and entinostat (0.5, 5, 50, 100 µmol/L) for 48 hours and observed a dose-dependent increase in CD26 expression on each myeloma cell by flow cytometry (Fig. 1C). Panobinostat-mediated increase in CD26 expression has been occurred at 0.5 nmol/L, whereas cytotoxicity against myeloma cells was not sufficient at this concentration. We observed a further increase in CD26 expression on myeloma cells following treatment with panobinostat at 5.0 to 50 nmol/L dose, correlated with dose-dependent enhanced anti-myeloma cytotoxic effect (Fig. 1C; Supplementary Fig. S1). Similarly, increasing concentrations of RG2833 or entinostat contributed to the enhanced expression levels of CD26 on myeloma cells at 5 nmol/L to 0.5 µmol/L dose of RG2833 as well as 0.5 to 50 µmol/L dose of entinostat. In parallel, more significant myeloma cell death was induced at 0.5 µmol/L dose of RG2833 and 50 µmol/L dose of entinostat. Although, higher doses of RG2833 at 5.0 µmol/L or entinostat at 500 µmol/L further reduced the viability of myeloma cells, these doses did not induce greater CD26 expression in myeloma cells anymore (Fig. 1C; Supplementary Fig. S1) Collectively, HDACi by both broad and isoform-selective inhibitors exposure-time dependently as well as dose-dependently induced the upregulation of CD26 protein expression on myeloma cells.

To verify whether upregulation of CD26 in each myeloma cell was induced by nonspecific effects due to drug-induced cell stress, we additionally examined the impacts of bortezomib or melphalan on CD26 modulation in each myeloma cell by flow cytometry. Indeed, neither agents altered the expression levels of CD26 in KMS11, 26, 27, 28, and RPMI8226, regardless of the duration of their treatment (Supplementary Fig. S2).

IHC analysis also revealed that myeloma cells that remained untreated with HDACi expressed low or slightly detectable level of CD26, whereas myeloma cells treated with each HDACi for 48 hours showed moderately or intensely stained CD26 expression (Fig. 1D). Subsequently, removal of the HDACi for 48 hours resulted in the expression levels of CD26 on myeloma cells to the reduced or near-pretreatment levels again (Fig. 1E).

Next, to assess the impact of HDACi on CD26mRNA transcription in myeloma cells, we performed qRT-PCR analysis to measure the expression of CD26mRNA in myeloma cell lines. In the current study, the cDNA of myeloma cells, preincubated for 48 hours with or without each HDACi, was used for qPCR amplification of CD26 with specific primers (Supplementary Table S2). The CD26mRNA levels in each myeloma cell line were significantly increased on treatment with each HDACi (Fig. 1F). These data demonstrated that the induction of CD26 protein in myeloma cells is paralleled with an increase in CD26 mRNA transcription and therefore occurs at the level of CD26 gene transcription. Moreover, ELISA analysis showed that increased DPPⅣ enzymatic activity in myeloma cells treated with each HDACi for 48 hours was correlated with the induction of CD26 protein in myeloma cells (Fig. 1G).

In addition, we analyzed whether each HDACi modulates the expression of other cell surface molecules used as therapeutic targets of multiple myeloma. The expression levels of CD38 were time-dependently increased in KMS11 on treatment with each HDACi excluding entinostat, whereas levels of CD38 were upregulated in KMS27 and RPMI8226 only on treatment with tubastatinA (Supplementary Fig. S3–S5). Moreover, BCMA expression was significantly upregulated in KMS27 and RPMI8226 treated with tubastatinA. SLAMF7/CS1 expression was also markedly enhanced in KMS11 and RPMI8226, treated with tubastatinA (Supplementary Fig. S3–S5).

### Synergistic Anti-myeloma Efficacy of HDACi plus Humanized Anti-CD26mAb Against CD26^neg^ Myeloma Cells

**FIGURE 2:**
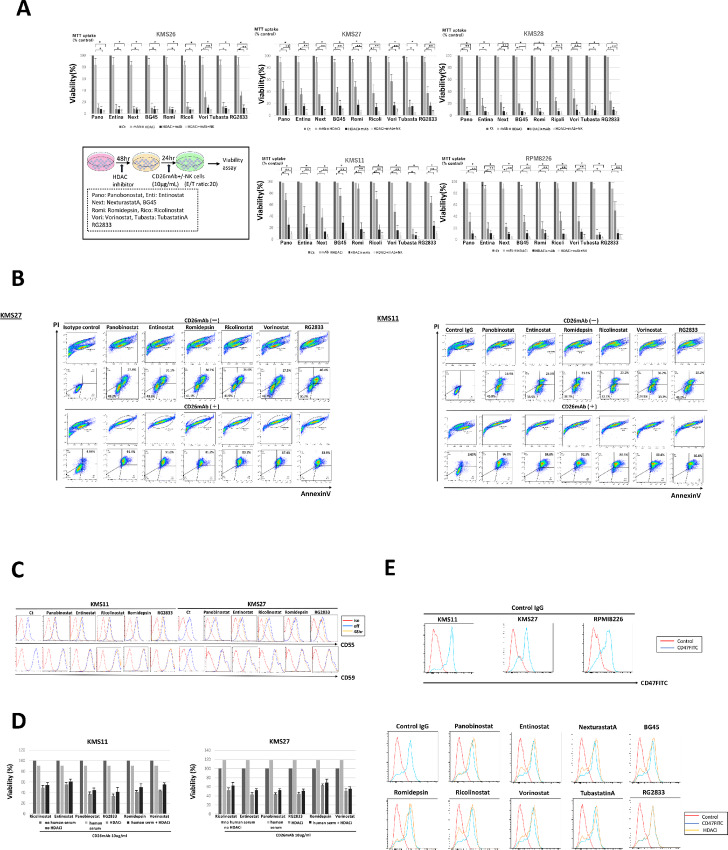
The effects of HDACi on the viability of CD26^neg^ myeloma cell lines treated with humanized anti-CD26 monoclonal antibody (CD26mAb). **A,** Cytotoxicity of CD26mAb against myeloma cell lines KMS11, 26, 27, 28, and RPMI8226 is shown with or without the treatment of HDACi. First, myeloma cells were pretreated with one of nine HDACi for 48 hours, followed by additional incubation with either CD26mAb at a concentration of 10 µg/mL or isotype control IgG_1_ for 24 hours in the presence or absence of human NK effector cells at an E/T ratio of 20:1 and then, the viability of target myeloma cells was analyzed by the luciferase assay. Bar diagrams, indicating the percentage of viable myeloma cells show that CD26mAb alone did not induce significant lysis of CD26^neg^ myeloma cells, whereas, the combination with HDACi plus CD26mAb synergistically facilitated lysis of CD26^neg^ myeloma cells via direct effects as well as via NK effector cell-mediated ADCC by mAb. The data represent the mean ± SE of triplicate wells from the representative of three independent experiments. The error bars represent the range, *, *P* < 0.05; **, *P* < 0.01. **B,** KMS11 and KMS27 contained residual viable cell populations, which existed (Annexin/PI) after monotherapy with one of six HDACi for 48 hours. In contrast, combination with each HDACi plus CD26mAb overcame this refractoriness and synergistically augmented lysis of CD26^neg^ cell populations in KMS11 and KMS27, which were refractory to HDACi or CD26mAb treatment as a single agent. **C,** Levels of CIPs (CD55, CD59) in myeloma cells in the presence or absence of one of five HDACi were examined using flow cytometry. Myeloma cells were positive for both CD55 and CD59 both before and after treatment with each HDACi. The expression levels of these proteins in myeloma cells underwent minor or no changes following treatment with each HDACi. **D,** The CDC lysis against CD26^neg^ myeloma cells on treatment with CD26mAb was examined after the exposure to one of five HDACi for 48 hours. Incubation of target myeloma cells was performed for 1 hour in the presence of human serum plus CD26mAb at a concentration of 10 µg/mL or control IgG_1_. No marked CDC lysis by CD26mAb was observed against myeloma cells, regardless of the pretreatment with each HDACi. **E,** The expression levels of CD47 in myeloma cell lines were examined by flow cytometry; KMS11, 27, and RPMI8226 were intensely stained with CD47. In addition, KMS11 was treated with each HDACi for 48 hours and the CD47 expression was also analyzed. Treatment with each HDACi resulted in no alterations in CD47 expression levels of KMS11.

We demonstrated that HDACi retains the ability to induce the increased expression levels of CD26 on CD26^neg^ myeloma cells, both at the mRNA and protein levels. Furthermore, we investigated the impact of HDACi on the viability of CD26^neg^ myeloma cells in the presence or absence of CD26mAb. We pretreated myeloma cell lines KMS11, 26, 27, 28, and RPMI8226 with one of nine HDACi for 48 hours. Thereafter, these cells were treated with CD26mAb at 10 µg/mL or isotype control IgG_1_ and additionally incubated for 24 hours in the presence or absence of human NK effector cells at an E/T ratio of 20:1 and then, the viability of each myeloma cell line was analyzed by antibody-dependent cellular cytotoxicity (ADCC) assay. The results showed that the treatment with each HDACi as a single agent induced significant myeloma cell death in KMS26 and KMS28, whereas KMS27 and KMS11 contained cell populations that were refractory to the treatment with each HDACi alone (Fig. 2A). Furthermore, although monotherapy with CD26mAb did not induce significant lysis of CD26^neg^ myeloma cells in any of myeloma cell line, treatment with HDACi plus CD26mAb in combination synergistically facilitated lysis of CD26^neg^ myeloma cells via direct effects as well as NK cell–mediated ADCC by CD26mAb and this combined treatment overcame the therapeutic refractoriness of CD26^neg^ myeloma cells to CD26mAb (Fig. 2A). In particular, the combination with HDACi plus CD26mAb readily augmented the lysis of CD26^neg^ cell populations in KMS27 or KMS11 that were refractory to treatment with HDACi or mAb alone (Fig. 2B).

To further explore the mechanisms that contribute to the refractoriness of myeloma cells toward CD26mAb therapy, we assessed the levels of complement inhibitory proteins (CIP) CD55 and CD59 in myeloma cells incubated with one of five HDACi for 48 hours. CIPs protect myeloma cells from a complement attack via complement-dependent cytotoxicity (CDC) by mAb (36); therefore, the elevated expression levels of CIPs observed in untreated myeloma cells indicate the inhibition of CDC by CD26mAb in myeloma cells. Moreover, the expression levels of these CIP proteins underwent only minor changes and were similar between HDACi-treated and nontreated cells (Fig. 2C). Consistent with our previous data (29), these results suggest that the induction of CD26 in myeloma cells by HDACi does not enhance CDC activity by CD26mAb against CD26^neg^ myeloma cells (Fig. 2D).

CD47 expression in myeloma cells also leads to immune evasion through its interaction with signal regulatory proteins on dendritic cells or macrophages (37). Therefore, CD47 blockade may offer a therapeutic approach for preventing the immune escape of tumor cells. Indeed, the basal expression levels of CD47 were high in KMS11, KMS27, and RPMI8226. Furthermore, CD47 expression levels in KMS11 were shown to be sustained, but revealed no changes on treatment with each HDACi; this finding may be associated with therapeutic refractoriness (Fig. 2E).

### Transcriptomic Alterations in Myeloma Cells, Treated with HDACi ± CD26mAb versus Untreated Cells

**FIGURE 3:**
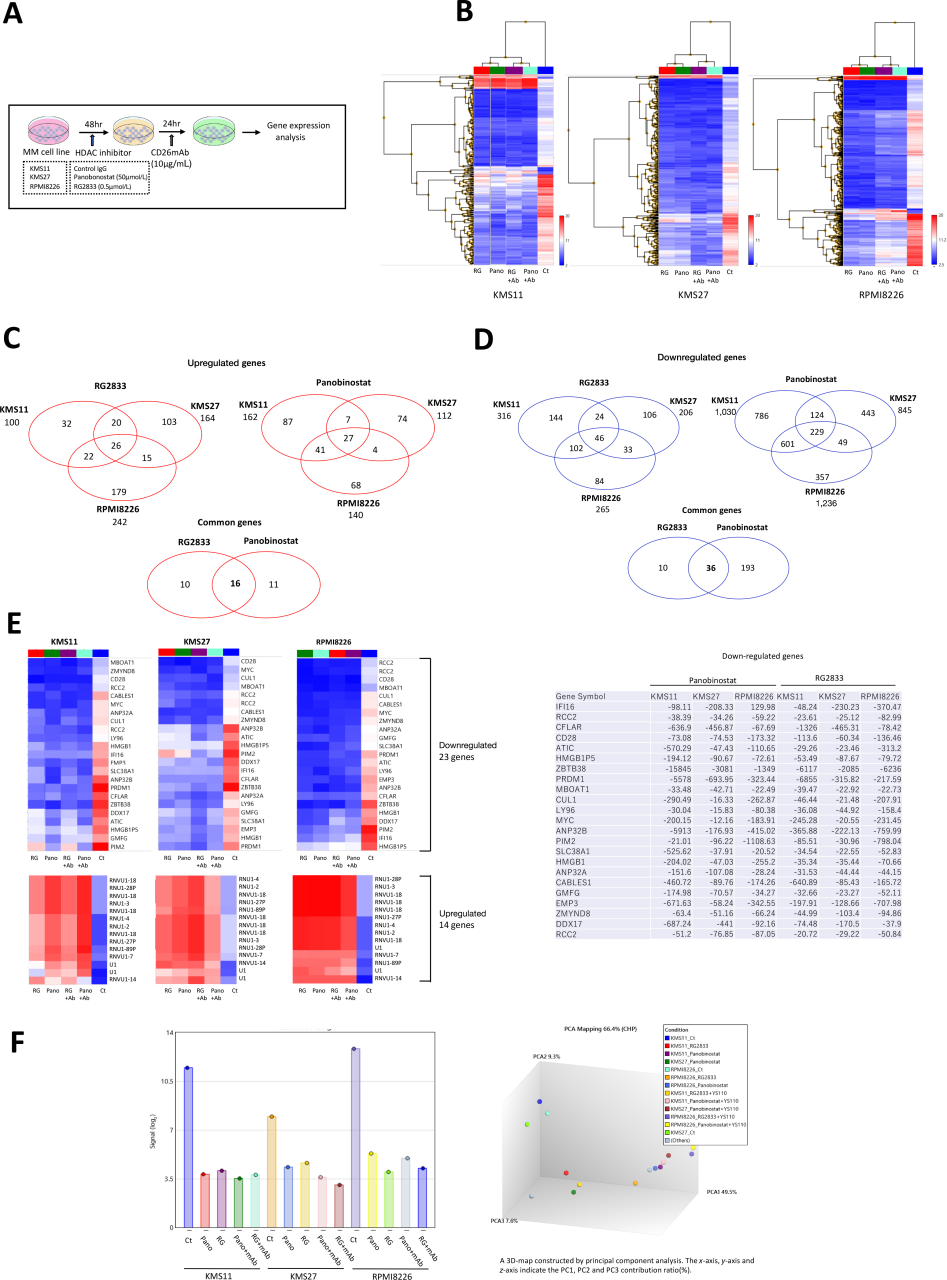
Transcriptomic profiles in three myeloma cell lines, either treated or untreated with HDACi or HDACi plus CD26mAb. **A,** Schema of transcriptomic analysis of three myeloma cell lines KMS11, 27, and RPMI8226, treated with one of two HDACi by panobinostat (50 µmol/L) or RG2833 (0.5 µmol/L) or HDACi plus CD26mAb in combination. **B,** Three myeloma cell lines were treated with panobinostat (50 µmol/L) or RG2833 (0.5 µmol/L) for 48 hours, followed by additional incubation with CD26mAb for 24 hours and transcriptomic profiles in each myeloma cell line were analyzed at each timepoints using microarray analysis. The heat maps show normalized relative mRNA expression differences, based on log_2_ fold change, with a cut-off *P* value <0.05. The color scale of the heat map from blue to red indicates low to high expression. **C,** Venn diagrams showing overlap in the most significantly upregulated genes (log_2_ fold change >10, with a cut-off *P* value <0.05) among three myeloma cell lines treated with either panobinostat (50 µmol/L) or RG2833 (0.5 µmol/L) for 48 hours compared with the isotype control IgG_1_. **D,** Venn diagrams showing overlap in the most significantly downregulated genes (log_2_ fold change <−10, with a cut-off *P* value <0.05) among three myeloma cell lines treated with either panobinostat (50 µmol/L) or RG2833 (0.5 µmol/L) for 48 hours compared with the isotype control IgG_1_. **E,** Among the 36 downregulated genes common to all three myeloma cell lines with the treatment of either panobinostat or RG2833, overlapped 23 genes excluding 13 noncoding genes were identified (log_2_ fold change <−10, with a cut-off *P* value<0.05). The values show the fold changes of mRNA expression of transcripts in each myeloma cell line treated with each HDACi or either HDACi plus CD26mAb compared with control IgG_1_. The color scale of the heat map from blue to red indicates low to high expression. Similarly, among the 16 genes, commonly upregulated in all three myeloma cell lines on treatment with each HDACi, overlapped 14 genes excluding noncoding genes were indicated. **F,** Left, c-Myc gene signal (log_2_) in KMS11, KMS27, and RPMI8226, treated with isotype control IgG_1_, HDACi; panobinostat or RG2833 and HDACi plus CD26mAb were shown. Myc is one of the genes, significantly downregulated in common, excluding noncoding genes in three myeloma cell lines following treatment with each HDACi or either HDACi plus CD26mAb. Right, A three-dimenisonal MAP was constructed by principal component analysis, indicating gene expression patterns based on transcriptome analysis.

To gain the insight into the mechanisms by which HDACi modifies myeloma cell function, we analyzed the transcriptomic profiles of three myeloma cell lines in response to treatment with HDACi by either broad or isoform-specific inhibitor in the presence or absence of CD26mAb, compared with control IgG_1_, further we identified the sets of genes with significantly altered expression levels. Briefly, KMS11, 27, and RPMI8226 were treated with panobinostat or RG2833 for 48 hours, followed by additional incubation with the CD26mAb for 24 hours and then, changes of mRNA expression levels in each myeloma cell line was explored at each timepoint (Fig. 3A). We showed that each myeloma cell line exhibited the majority of changes relative to control IgG_1_ in the expression levels of mRNA transcripts following treatment with each HDACi, regardless of the presence or absence of CD26mAb (Fig. 3B). Transcriptomic profiles revealed that 27 and 26 genes, respectively were commonly upregulated in all three myeloma cell lines following treatment with panobinostat or RG2833. Of these genes, 16 genes were most significantly upregulated and shared by both HDACi (log_2_ fold change > 20 to the control with *P* < 0.05; Fig. 3C). On the other hand, 229 and 46 genes, respectively were commonly downregulated in all three myeloma cell lines following treatment with panobinostat or RG2833. Of these genes, 36 genes were most significantly downregulated and sheared by both HDACi (log_2_ fold change <−10) to the control with *P* value < 0.05; Fig. 3D). Moreover, of these overlapped 36 genes, 23 genes with consistently decreased expression levels were identified excluding 13 noncoding genes (log_2_ fold change <−10) to the control with *P* value < 0.05; Fig. 3E; Supplementary Table S3). These results suggest that myeloma cells treated with HDACi alone as well as with HDACi plus CD26mAb in combination exhibited similar and distinct gene expression signatures, compared with those treated with control IgG_1_. Furthermore, the downregulated genes shared among the three myeloma cell lines and treatment with both HDACi contained factors involved in cell cycle regulation, cell proliferation, cell differentiation, and apoptosis of cells such as Myc and Pim-2: the inhibition of which is associated with cell cycle arrest and apoptosis of myeloma cells (refs. 38–40; Fig. 3E and F). Notably, the majority of human cancers present with overexpression of Myc, which we also validated using qPCR analysis. Indeed, Myc expression affects gene instability and tumorigenesis via the activation or repression of a number of target genes as well as by the regulation of the gene promoter (38, 39). In addition, Myc expression is reportedly further upregulated during the course of disease progression in multiple myeloma (41). Therefore, next we sought to explore whether Myc regulation may contribute to the CD26 induction of in myeloma cells by HDACi.

### The 5′-flanking Region of the Human CD26 Gene

**FIGURE 4:**
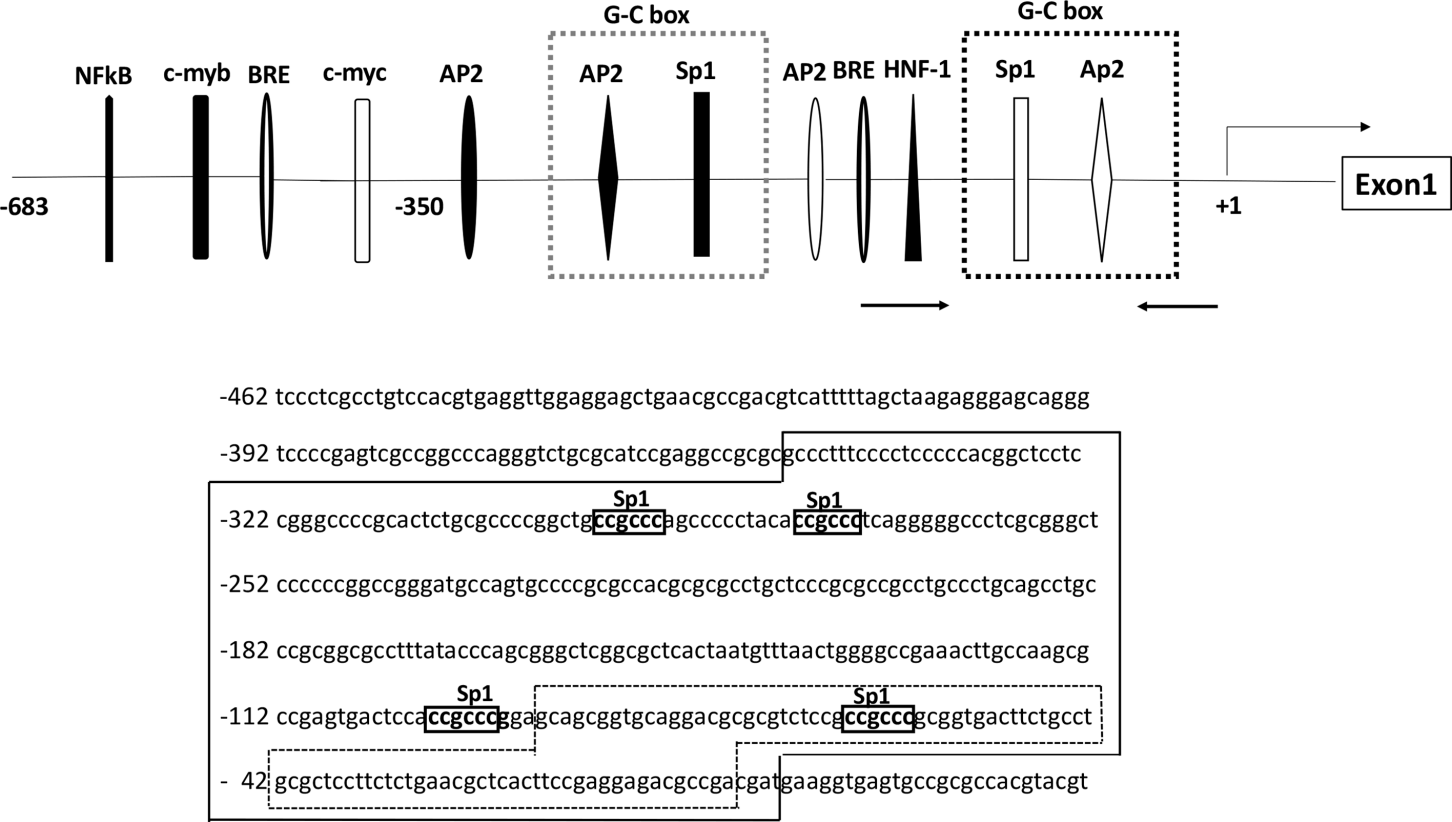
The 5′-flanking region of the human CD26 gene. The schematic representation of the 5′-flanking region of the human CD26 gene promoter constructs and the associated nucleotide sequences is shown. The 5′-flanking region of the CD26 gene contains 300 bp (−359 to +1) and incorporates potential binding sites for several transcriptional factors including G-C boxes for the binding of Sp1 and AP2, BRE, and HNF, the relative locations of which are indicated by the symbols in the figure. In particular, 89 bp of the G-C rich region (−91 to −3 relative to the translation initiation site) in the proximal 5′-flanking region is essential for CD26 promoter activity. The nucleotide sequences including the 5′-flanking region of the CD26 promoter are also shown. Position +1 indicates the initiation of the coding region in exon1. The nucleotide sequence has been shown in the GeneBank database with the accession number of AH005372.

The human CD26 gene, located on chromosome 2 (2q24.3): contains 300 bp of the 5′-flanking region (−359 to +1) which includes potential binding sites for several transcriptional factors related to cell proliferation and differentiation such as Sp1 (specificity protein 1), Ap2, BRE (butyrate-responsive element), and HNF (hepatic nuclear factor 1; refs. 42, 43; Fig. 4). In particular, the 89 bp of G-C rich region (−91 to −3 relative to the translation initiation site), located at the proximal 5′-flanking region just upstream of the transcription initiation site, is essential for CD26 promoter activity (ref. 44; Fig. 4).

Sp1 is a transcription factor that is expressed in several solid tumor cells as well as in myeloma cells. Overexpression of Sp1 is involved in tumor progression or metastasis via regulation of the expression of Sp1-responsive genes related to cell growth, apoptosis, and angiogenesis (45, 46). These genes contain G-C rich regions, that is, G-C boxes on the promoter that interacts with Sp1 (47). Indeed, the CD26 gene contains G-C boxes for Sp1 binding at both the proximal and distal regions within the promoter which regulate the expression of proto-oncogenes such as Myc, ras, and pim-1 (refs. 42, 43; Fig. 4). Furthermore, c-Myc not only affects proliferation, apoptosis, and metabolism in tumor cells via its modification but also forms complexes with Sp1 on several promoters and titrates the levels of Sp1, thereby affecting the promoter activity of several genes (47). Consequently, we postulated that modulation of c-Myc may play several roles in regulation of the CD26 promoter in myeloma cells. We therefore sought to elucidate the epigenetic impacts of histone as well as c-Myc as non-histone on the CD26 promoter of myeloma cells in the absence or presence of HDACi and determine whether it results in the induction of CD26 expression in myeloma cells.

### HDACi Acetylates Histone 3 Protein on the CD26 Promotor Region of Myeloma Cells

**FIGURE 5:**
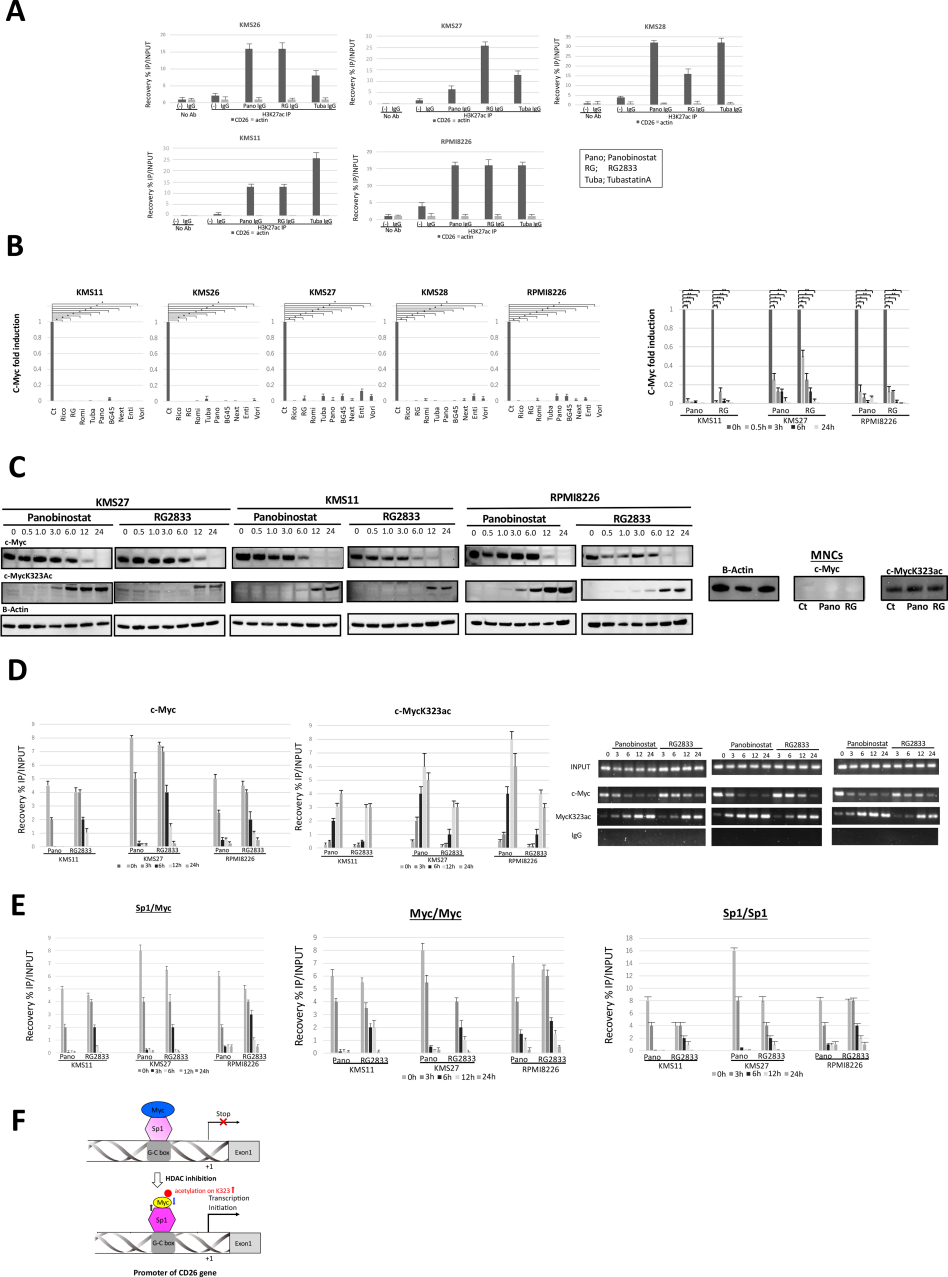
Epigenetic modification at the CD26 promoter of myeloma cells on treatment with HDACi. **A,** The effects of HDACi on histone H3 acetylation on the CD26 promoter of myeloma cells are shown. Myeloma cell lines KMS11, 26, 27, 28, and RPMI8226, treated with either panobinostat (50 µmol/L), RG2833 (0.5 µmol/L), or tubastatinA (2.5 µmol/L) or control IgG_1_ for 48 hours were investigated by ChIP assay using anti-histone 3 on lysine 27 acetylated (H3K27Ac) antibody or rabbit IgG and then, the DNAs of immunoprecipitated chromatin were amplified and quantified by real-time qPCR with the primer pairs for the CD26 promoter shown in Supplementary Table S1. NoAb means samples prepared without antibodies as a control and INPUT indicates that PCR was performed with genomic DNA. Increased levels of acetylation at H3K27 was observed on the CD26 promoter of myeloma cells treated with each HDACi, compared with control IgG_1_. Values represent percentage of IP/INPUT. Bars represent the mean ± S.D. of three independent experiments done in triplicate. **B,** qRT-PCR for c-Myc expression in myeloma cell lines KMS11, 26, 27, 28, and RPMI8226 treated with one of nine HDACi, namely, BG45 (1 µmol/L), romidepsin (0.125 µmol/L), ricolinostat (0.5 µmol/L), panobinobinostat (50 µmol/L), entinostat (50 µmol/L), nexturastatA (0.125 µmol/L), vorinostat (1 µmol/L), tubastatinA (25 µmol/L), RG2833 (0.5 µmol/L), or control IgG for 48 hours. The expression of c-Myc mRNA was significantly reduced in each myeloma cell line after exposure to each HDACi for 48 hours (*, *P* < 0.01). Furthermore, the expression levels of c-Myc mRNA in KMS11, 27, and RPMI8226 were examined at the indicated times (3.0, 6.0, 12, 24 hours) following treatment with panobinostat or RG2833 (*, *P* < 0.05; **, *P* < 0.01). The expression levels of c-Myc were normalized to that of GAPDH and quantified by the 2^−△△Ct^ method. Data are shown as the ratio of c-Myc mRNA/GAPDH mRNA and represent the means ± S.D. of three independent experiments. **C,** Expression levels of c-Myc and c-MycK323ac protein in KMS11, 27, and RPMI8226 were examined in the presence or absence of panobinostat (50 µmol/L) or RG2833 (0.5 µmol/L) at the indicated times (0.5, 1.0, 3.0, 6.0, 12, 24 hours) by immunoblotting. Levels of these proteins in normal MNCs, incubated with panobinostat, RG2833 or control IgG_1_ for 24 hours were also analyzed. **D,** To investigate the binding of c-Myc and c-MycK323ac to the CD26 promoter of myeloma cells, KMS11, 27, and RPMI8226 were treated with panobinostat (50 µmol/L), RG2833 (0.5 µmol/L), or control IgG_1_ for the indicated times (3.0, 6.0, 12, 24 hours) and then, ChIP assays were conducted in each myeloma cell using anti-c-Myc or c-MycK323ac antibody or rabbit IgG. Thereafter, the DNAs of each immunoprecipitated chromatin suspension were amplified and quantified by real-time qPCR with specific primers for the CD26 promoter via the proximal G-C box (Supplementary Table S1). The recovery of ChIP's DNAs was calculated as the percentages of IP/INPUT. The time-dependent decrease of the binding of c-Myc to the promoter, concomitant with a time-dependent increase in the binding of c-MycK323ac to the promoter, was observed in each myeloma cell line treated with each HDACi, compared with control IgG_1_. Bars represent the mean ± S.D. of three independent experiments done. The amplified products were also visualized by MIDORI^green^ Direct staining following 1.5% agarose gel electrophoresis. Representative data of 40 cycles are shown. INPUTs show that PCR was conducted with genomic DNA. The actin signal shows equal loading as a control. **E,** The binding of c-Myc to the transcriptional factor, Sp1 in myeloma cell lines is shown after exposure to each HDACi. The binding of c-Myc to the Sp1 on the CD26 promoter via the proximal G-C box of KMS11, 27, and RPMI8226 after exposure to panobinostat (50 µmol/L) or RG2833 (0.5 µmol/L) was examined by ChIP and re-ChIP assay using antibodies for c-Myc and Sp1, followed by real-time qPCR using specific primers to amplify the CD26 promoter, including the proximal G-C box. The recovery of ChIP's DNAs was calculated as the percentages of IP/INPUT for each sample. In the absence of HDACi, c-Myc binds to the Sp1 on the proximal G-C box of the CD26 promoter in each myeloma cell line, whereas in the presence of HDACi, this binding was time-dependently detached. Bars represent the mean ± S.D. of three independent experiments. **F,** Schema of HDACi-regulated CD26 induction in myeloma cells.

First, we examined the epigenetic status in histone protein on the CD26 promoter region of myeloma cells after treatment with HDACi. ChIP assays were conducted on five myeloma cell lines, either treated or not treated with one of three HDACi, that is, panobinostat, RG2833 and tubasatinA, for 48 hours. The DNAs of immunoprecipitated chromatin of each myeloma cell line were analyzed using real-time qPCR with specific primers to amplify the CD26 promoter region (Supplementary Table S1). It was demonstrated that an increased levels of acetylation in histone 3 lysine 27 (H3K27) was detected on the CD26 promoter of each myeloma cell line after exposure to HDACi, suggesting its part of roles in the mechanisms involved in HDACi-dependent CD26 promoter activation in myeloma cells (Fig. 5A).

### c-Myc Binds to the CD26 Promoter of Myeloma Cells Through Sp1 on the Proximal G-C Box

Next, we explored the epigenetic impacts of c-Myc on the CD26 promoter of myeloma cells and determined whether these contributed to the induction of CD26 expression in myeloma cells. We first examined the expression levels of c-Myc mRNA in five myeloma cell lines following the treatment with one of nine HDACi (Fig. 5B) and the significant reductions in mRNA expression levels of each myeloma cell were validated after 48 hours of exposure to each HDACi. Subsequently, the time course changes in c-Myc expression levels in KMS11, 27, and RPMI8226 with or without treatment by panobinostat or RG2833, were assessed in detail using real-time qPCR and immunoblotting (Fig. 5B and C). The expression levels of c-Myc were exposure time-dependently decreased both at mRNA and protein levels in myeloma cell lines on treatment with each HDACi (Fig 5B and C). Moreover, c-Myc was still significantly downregulated after 12 to 24 hours of exposure to each HDACi, which was fully consistent with the transcriptomic profiles of myeloma cell lines in the presence or absence of HDACi, as shown in Fig. 3E and F. Furthermore, the addition of cycloheximide did not restore HDACi-mediated c-Myc reduction in myeloma cells, implying that the c-Myc expression is regulated in myeloma cells not only transcriptionally and translationally but also posttranslationally (Supplementary Fig. S6). In addition, the acetylation status of c-Myc in each myeloma cell line was investigated in the presence or absence of each HDACi. The acetylation of c-Myc on lysine 323 (K323Ac) in each myeloma cell line was evident after 3 to 6 hours of exposure to panobinostat and was further increased after 12 to 24 hours of its exposure. It was also potentiated after 12 to 24 hours of the exposure to RG2833 (Fig. 5C). These results indicated that the expression levels of c-MycK323ac were time-dependently increased in myeloma cells after treatment with each HDACi and were inversely correlated with the time-dependent decrease in c-Myc expression (Fig. 5C). On the other hand, expression levels of both c-Myc and acetylated c-Myc were unaltered in normal human mononuclear cells on treatment with HDACi, implying that c-Myc regulation in myeloma cells is a tumor-specific process (Fig. 5C).

We further investigated the interaction between c-Myc and the CD26 promoter in myeloma cells with or without treatment by HDACi. ChIP analysis was conducted in KMS11, 27, and RPMI8226 in the presence or absence of panobinostat or RG2833 at the indicated times (3.0, 6.0, 12, 24 hours) using antibodies for c-Myc and c-MycK323ac. Thereafter, DNAs of immunoprecipitated chromatin in each myeloma cell line were subjected to qPCR to amplify the CD26 promoter region, including the proximal G-C box (Supplementary Table S1). It was revealed that the recovery percentages of IP/INPUT, indicating the binding of c-Myc to the CD26 promoter via the proximal G-C box was exposure time-dependently decreased on treatment with HDACi, whereas the binding of c-MycK323ac to the promoter showed a time-dependent increase in each myeloma cell line following treatment with HDACi. These findings suggest that the occupancy of c-Myc was replaced by that of c-MycK323ac on the CD26 promoter of myeloma cells on treatment with HDACi (Fig 5D).

Finally, on the basis of these observations, we further examined the interaction between c-Myc and Sp1 on the CD26 promoter of myeloma cells in the presence or absence or HDACi. DNAs of chromatin, immunoprecipitated by ChIP and re-ChIP assay using antibodies for Sp1 and c-Myc in each myeloma cell line, either treated or untreated with each HDACi for the indicated times (3.0, 6.0, 12, 24 hours), were subject to qPCR and amplified at the CD26 promoter, including the proximal G-C box (Supplementary Table S1). The recovery percentages of IP/INPUT, indicating the binding of c-Myc to the Sp1 was exposure time-dependently reduced on the CD26 promoter of myeloma cells on treatment with each HDACi (Fig. 5E).

These results suggest that in the absence of HDACi, c-Myc is attached to the CD26 promoter via binding to Sp1 and thereby represses the promoter, leading to interruption of CD26 transcription in myeloma cells. In contrast, in the presence of HDACi, c-Myc is detached from the CD26 promoter via Sp1 with the increased acetylation of c-MycK323 and the promoter is thereby activated, leading to initiation of CD26 transcription as well as activation of cytotoxicity in several myeloma cells (Fig 5F).

### The Effect of HDACi and CD26mAb on the Activity of Human NK Cells in Multiple Myeloma

NK cells are crucial mediators of ADCC against myeloma cells by mAb therapy targeting CD38, CS1, and BCMA in multiple myeloma. Moreover, our previous study revealed that IMiDs potentiated human NK cell activity, leading to enhanced ADCC by CD26mAb against CD26^pos^ myeloma cells (29).

**FIGURE 6:**
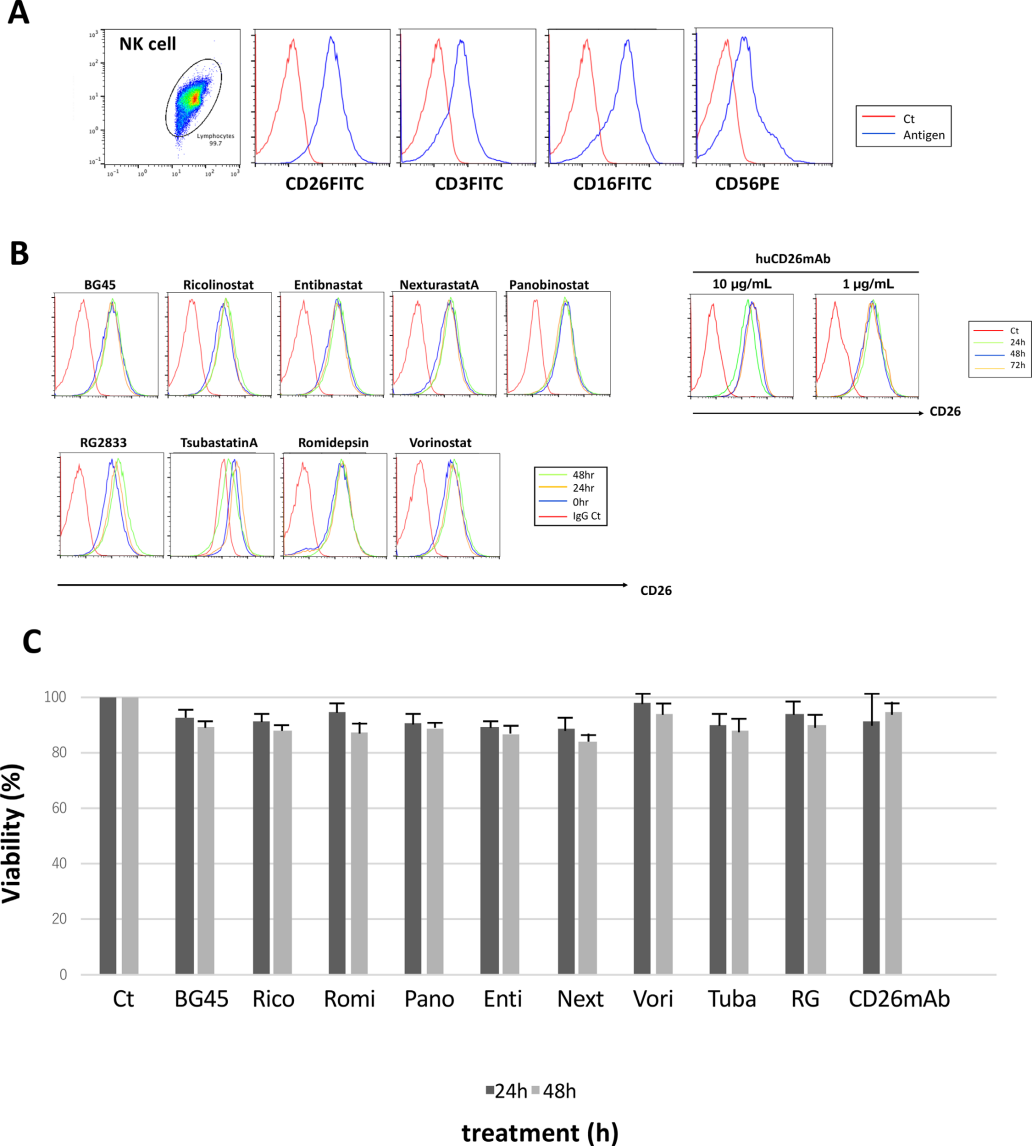
The effect of HDACi and CD26mAb on human NK cells in multiple myeloma. **A,** Expression of CD26, CD3, and CD56 on human NK cells was examined by flow cytometry. Representative overlay histograms show that human NK cells were positive for CD26. **B,** Expression levels of CD26 on human NK cells were investigated before and after exposure to one of nine HDACi or CD26mAb at 10 µg/mL for indicated times (24, 48, 72 hours). Representative overlay histograms show the expression levels of CD26 on NK cells: these levels were not altered following treatment with each HDACi. **C,** After NK cells were treated with one of nine HDACi or CD26mAb at 10 µg/mL for 24 and 48 hours, the viability of NK cell was examined by MTT assay. Bar diagrams show the percentage of viable cells. Data are presented as mean values ± S.D of three independent experiments.

To clarify the effect of HDACi or CD26mAb on NK cell activity in multiple myeloma, the expression levels of CD26 in NK cells in the presence or absence of one of nine HDACi was explored. Flow cytometry analysis showed that human NK cells exhibited high expression levels of CD26: these expression levels were not significantly affected by exposure to either HDACi or CD26mAb for the indicated times (24, 48, 72 hours; Fig. 6A and B). Furthermore, the effects of HDACi or CD26mAb on the viability of NK cells were assessed using MTT assay. Our findings demonstrated that although both HDACi and CD26mAb moderately affected the viability of NK cells, its viability did not show significant change, implying that ADCC activity against myeloma cells is likely not severely disrupted by treatment with HDACi plus CD26mAb in combination.

## Discussion

Recent development of novel antibody and cellular-based therapies, directly targeting antigens such as BCMA on myeloma cells has resulted in durable responses in patients with RRMM (8–18). Our previous studies demonstrated favorable preclinical results showing potent *in vitro* and *in vivo* cytotoxic efficacy of CD26mAb against both CD26^pos^ myeloma cells and CD26^pos^ osteoclasts in multiple myeloma (28, 29). However, BM tissues of patients with multiple myeloma revealed heterogeneous and sometimes decreased expression levels of CD26 in plasma cells in contrast to osteoclasts in which CD26 is uniformly and intensely expressed (refs. 28, 29; Fig. 1). These results might indicate barriers to elicit robust responses in multiple myeloma by CD26mAb.

In the current study, we demonstrated that epigenetic regulation by HDACi in myeloma cells, especially isoform-selective inhibition of HDAC1, 2, 3, and 6 as well as broad inhibition induces an increase in CD26 transcripts by RT-PCR and CD26 protein expression by flow cytometry and IHC. Therefore, concurrent use of HDACi conferred superior cytotoxic efficacy of CD26mAb against CD26^neg^ myeloma cells or those with CD26 antigen loss. Consequently, we further elucidated the precise mechanisms involved in the induction of CD26 expression in myeloma cells by HDACi.

Comparison of the transcriptomic profiles of three myeloma cell lines between those treated with HDACi by broad or isoform-selective inhibitor and those left untreated identified Myc as one of the aberrantly deregulated genes, which is known to be a hallmark in the majority of human cancers: it regulates cell proliferation, differentiation, and apoptosis via regulation of a number of target genes and is involved in tumorigenesis (38, 48, 49). HDACs are known to have majority of substrates including histone and non-histone proteins which are involved in biological processes such as cell proliferation, differentiation, apoptosis, and cell death. In particular, non-histone proteins regulate the activity of tumor suppressor genes and oncogenes which have crucial roles in tumorigenesis (30, 31). Therefore, HDACi induce different phenotypes in tumor cells and antitumor effects via cell cycle arrest, activation of apoptosis, mitotic cell death, autophagic cell death, and reactive oxygen species–induced cell death (30, 31). The c-Myc is one of the non-histone proteins as a substrate of HDACs, which is also subject to posttranslational modifications such as phosphorylation, acetylation, and ubiquitinylation (39). It plays crucial roles in the development of plasma cell malignancies during the progression from monoclonal gammopathy of unknown significance to smoldering multiple myeloma, symptomatic multiple myeloma, and plasma cell leukemia (41, 48, 49). Moreover, BM microenvironment upregulates c-Myc, thereby promoting myeloma cell growth. Therefore, Myc may become an attractive therapeutic target for the treatment of multiple myeloma (48, 49). The c-Myc is strongly modulated (i.e., downregulated or acetylated) by HDACi, which correlates with cell cycle arrest and apoptosis of cells via restoring the expression of genes aberrantly repressed in tumor cells, leading to tumor reduction (38, 48, 49). Indeed, in the current study, we validated the time-dependent reduction of c-Myc as well as an increase of c-MycK323ac in myeloma cell lines on treatment with class I/II and class I HDACi, compared with those treated with control IgG_1_ at protein levels (Fig. 5C), but not in human normal MNCs, highlighting that c-Myc modification by HDACi is tumor-selective and may correlate with cytotoxic response in myeloma cells.

Our results demonstrated that the cell surface CD26 expression in KMS26, 27, 28 and RPMI8226 was upregulated in parallel to anti-myeloma cytotoxicity on treatment with HDACi. On the other hand, despite the upregulation of CD26 expression in KMS11, its cytotoxic effect was not sufficiently clear following treatment with several class Our results demonstrated that the cell surface CD26 expression in KMS26, 27, 28, and RPMI8226 was upregulated in parallel to anti-myeloma cytotoxicity on treatment with HDACi. On the other hand, despite the upregulation of CD26 expression in KMS11, its cytotoxic effect was not sufficiently observed following treatment with several class I/II or class I HDACi, suggesting that the upregulation of CD26 in myeloma cells was not necessarily correlated with anti-myeloma cytotoxicity by HDACi. Moreover, our findings have important clinical implications indicating that modest HDACi contributes to synergistic anti-myeloma cytotoxicity by CD26mAb. Namely, potent HDACi may trigger adverse reactions, thereby compromising the combination with HDACi plus CD26mAb for the treatment of multiple myeloma.

The promoter region of the CD26 gene contains several potential transcription factor binding sites including Sp1 (Fig 4) and this site is shown to be a potent transcriptional activator for the transcription of several genes (42–46). The c-Myc reportedly binds to the DNA-binding domain of Sp1 on the promoter of several genes and titrates the levels of Sp1, thereby affecting promoter activity (47). Intriguingly, the c-Myc gene contains multiple Sp1-binding sites within its promoter, and represses its own transcription through the interaction between c-Myc and Sp1 at the promoter (47, 48, 50). Therefore, we postulated that the interaction between c-Myc and Sp1 on the CD26 promoter of myeloma cells may be one of mechanisms which regulate the activity of the CD26 promoter gene to induce CD26 expression in myeloma cells. ChIP-qPCR analysis confirmed that in the absence of HDACi, c-Myc is present on the CD26 promoter of myeloma cells via binding to Sp1 located on the proximal G-C box, thereby repressing the promoter and leading to reduced CD26 transcription in myeloma cells (Fig. 5D and E). In contrast, in the presence of HDACi, c-Myc was shown to be dissociated from Sp1 and its binding was replaced by acetylated c-Myc on K323 on the CD26 promoter of myeloma cells, leading to activation of the promoter and initiation of CD26 transcription (Fig. 5D and E).

The current study elucidated crucial roles of HDACi in the induction of CD26 expression in myeloma cells. First, class I or class II HDACi triggers modification of c-Myc in myeloma cells, associated with cytotoxicity in several myeloma cells. Second, HDACi plays the role of chemosensitizer via the induction of CD26 expression in myeloma cells with CD26 antigen loss. It results from increased acetylation of H3K27 as well as c-MycK323 on the CD26 promoter of myeloma cells and is inversely correlated with the decreased acetylation and expression of c-Myc. These changes lead to elicit superior cytotoxic efficacy of CD26mAb against CD26^neg^ or CD26^dim^ myeloma cells and restore refractoriness of myeloma cells to CD26mAb.

Currently, several HDAC inhibitors have received regulatory approval for solid tumors or hematologic malignancies including multiple myeloma (51). In particular, panobinostat have emerged as the only HDACi approved for the treatment of multiple myeloma which nonselectively inhibits class I/Ⅱ HDACs. HDACi has already shown synergistic antitumor effects with antitumor agents in combination. Indeed, the phase III PANORAMA1 trial demonstrated that 3-drug regimen containing panobinostat in combination with bortezomib plus dexamethasone led to a modest OS benefit, compared with 2-drug regimens containing bortezomib plus dexamethasone in patients with RRMM (52). Moreover, the combination of HDACi with immunotherapy has also been expected to reveal a dual efficacy as double epigenetic options. However, nonselective class I/II pan HDACi reveals profound anti-myeloma efficacy, whereas its clinical utility is limited because of unfavorable toxicities due to the inhibition of the broad range of HDAC isoforms (31, 51). Indeed, panobinostat causes severe toxic reactions such as BM suppression, severe diarrhea, bleeding tendency, liver or renal dysfunction, arrhythmia, and deep vein thrombosis, all of which were serious for elderly patients with multiple myeloma, resulting in the high rates of discontinuation in its treatment (31, 51). Therefore, alternatively, isoform-selective HDACi that exploits anti-myeloma cytotoxicity, while minimizing toxicities or combination regimens have recently been developed as a promising therapeutic option to improve the outcome of patients with multiple myeloma (53). Our findings also confirmed that isoform-selective (class I or class IIb) HDACi plus CD26mAb in combination may induce synergistic cytotoxicity against myeloma cells via the upregulation of CD26 on myeloma cells and enhanced ADCC activity by CD26mAb. In particular, HDAC3 has been reported to regulate c-Myc protein levels, thereby HDAC3 inhibition increased acetylation of c-Myc as well as DNMT1 in myeloma cells, leading to degradation of DNMT1 and inhibition of myeloma cell growth (54). Therefore, combination with HDAC3-selective inhibitor plus CD26mAb may be a promising therapeutic option to induce enhanced myeloma cell growth by CD26mAb.

To date, several studies have demonstrated that the efficacy of targeted immunotherapies in hematologic malignancies is partly dependent on the expression levels of the target antigen on the surface of tumor cells. Indeed, CD20^high^ malignant lymphoma cells or chronic lymphocytic leukemia cells potentiate cytotoxic efficacy of rituximab via CDC and ADCC, whereas CD20^low^ lymphoma or leukemia cells elicit a poor response to these mAbs (55, 56). Similarly, in multiple myeloma, the expression levels of CD38 in myeloma cells determine the efficacy of CD38 mAb-mediated cytotoxicity (34, 35). Specifically, CD38^high^ myeloma cells were rapidly eliminated by daratumumab via immune selection, indicating that the remaining myeloma cells had lower CD38 expression levels. In addition, the trogocytic transfer of complexes consisting of CD38 and daratumumab from the myeloma cell surface to immune effector cells is an additional important mechanism for CD38 antigen loss on both myeloma cells and immune cells. These processes reduce the therapeutic efficacy of daratumumab-mediated ADCC and CDC in multiple myeloma (57). Moreover, although BCMA is expressed on most of malignant plasma cells and is recognized as a validated target in multiple myeloma therapy, its expression levels are heterogeneous, resulting in variable responses in patients with multiple myeloma (5, 58). To date, BCMA antigen loss in myeloma cells remains poorly understood. Indeed, BCMA loss in myeloma cells is not common on treatment with anti-BCMA immunotherapies because BCMA is essential for the survival of malignant plasma cells (59). Moreover, the majority of cases of BCMA loss occur by immune selection after anti-BCMA targeted immunotherapies, and the expression levels of BCMA in myeloma cells recover to the pretreatment levels at a later time. Consequently, sequential anti-BCMA retreatment with different BCMA-directed immunotherapies is considered to be feasible (60).

The relation between CD26 expression and the efficacy of CD26mAb against CD26^pos^ malignancies has been controversial. Indeed, our previous studies demonstrated that CD26mAb revealed significant ADCC against CD26^pos^ myeloma cells but not against CD26^neg^ myeloma cells *in vitro* and *in vivo* (29). Likewise, in the treatment of solid tumors, Inamoto and colleagues showed that CD26mAb had inhibitory effects against CD26^pos^ MPM cells *in vitro* and exhibited antitumor effects in a CD26^pos^ MPM-bearing mouse model (61, 62). Consistent with preclinical results, CD26mAb has already been indicated as a promising therapy with well-tolerated toxicity profiles and modest efficacy as a single agent among patients with advanced MPM (26, 27); however, substantial differences in treatment response were also indicated against MPM. Indeed, a phase II study of CD26mAb in relapsed or refractory Japanese patients with MPM demonstrated that several cases with low CD26 expression in MPM cells could attain SD after treatment with CD26mAb (27). This finding implies that anti-MPM cytotoxicity may also be associated with mechanisms of action other than ADCC activity. In other words, refractoriness to targeted immunotherapies is not solely explained by antigen loss, but additional tumor- or host-related mechanisms underlying acquired resistance are also involved. First, an increase in the expression of CIPs, including CD55 and CD59, protects myeloma cells from complement attack via CDC, leading to refractoriness to mAb (36). Indeed, expression levels of CIPs on myeloma cell lines, cultured alone were increased but were not altered by HDACi, which may contribute to the inhibition of CDC lysis by CD26mAb in multiple myeloma, regardless of the presence or absence of HDACi (Fig. 2C and D). Second, myeloma cells reside in the BM microenvironment by binding to various stromal cells (BMSCs) through the upregulation of antiapoptotic proteins, which may also contribute to the development of resistance via the evasion of mAb or cytotoxic T cell–mediated killing of myeloma cells (63). We previously demonstrated that CD26mAb impaired the adhesion of CD26^pos^ myeloma cell to BMSCs which inhibits myeloma cell growth (29). Third, mAbs are not necessarily capable of eliminating clones in myeloma cells with high-risk chromatin alteration such as t(4;14)(p16.3;q32.3), gain 1q21 or del(17p) or drug-efflux pump such as side population (SP) cells, both of which indicate refractoriness to anticancer agents and result in inferior impacts on survival of patients with myeloma. Specifically, KMS11 cells contain both t(4;14) alteration and SP cells, parts of which revealed resistance to HDACi monotherapy; however, it was restored by treatment with HDACi plus CD26mAb in combination (Fig. 2A). Moreover, the activity of immune effector cells is also associated with refractoriness to targeted immunotherapy. CD38 is expressed not only on myeloma cells but also on NK cells. Therefore, rapid depletion of CD38^pos^NK cells was observed after treatment with daratumumab, which may lead to a decrease in the ADCC activity of daratumumab against myeloma cells (64, 65). In the current study, although CD26 was also highly expressed in NK cells, neither HDACi nor CD26mAb altered the expression levels of CD26 on NK cells (Fig. 6B). Moreover, the viability of NK cells was not affected by either treatment. This finding implies that NK cell–mediated ADCC against myeloma cells by CD26mAb is not impeded by the diminished frequency or activity of NK cells, unlike CD38mAb treatment, which may indicate a clinical benefit for the treatment of multiple myeloma by CD26mAb (Fig. 6C).

In summary, to overcome both innate and acquired refractoriness of myeloma cells with CD26 antigen loss to CD26mAb, the concurrent use of HDACi confers therapeutic benefit by the induction of CD26 expression in myeloma cells. Importantly, our findings point to a novel observation on the role of HDACi. Namely, epigenetic modification with isoform-selective (class I or class IIb) HDACi not only shows anti-myeloma activity in itself but also acts as a chemosensitizer by resensitizing CD26^neg^ myeloma cells or those with CD26 antigen loss to CD26mAb, thereby eliciting superior anti-myeloma cytotoxicity that may lead to restore the refractoriness to mAb in RRMM.

## Supplementary Material

Supplementary Methods S1 Supplementary Methods S1 include details of protocols for immunophenotyping (Supplementary Table S2), quantitation and qualification of mRNA levels, immunohistochemistry, immunoblotting, enzyme-linked immunosorbent assay (ELISA), and ChIP-qPCR.

Supplementary Figure S1 Supplementary Figure S1 shows effects of bortezomib or melphalan on CD26 expression on myeloma cells.

Supplementary Figure S2 Supplementary Figure S2 shows viability of KMS27 and KMS28, untreated or treated with panobinostat, RG2833 and entinostat at the indicated doses for 48 hours by MTT assay.

Supplementary Figure S3 Supplementary Figure S3 shows effects of HDAC inhibition on CD38 expression on myeloma cells.

Supplementary Figure S4 Supplementary Figure S4 shows effects of HDAC inhibition on BCMA expression on myeloma cells.

Supplementary Figure S5 Supplementary Figure S5 shows effects of HDAC inhibition on SLAMF7/CS1 expression on myeloma cells.

Supplementary Figure S6 Supplementary Figure S6 shows immunoblotting for c-Myc in KMS11, 27 and RPMI82226, treated with panobinostat or RG2833 in the presence or absence of cycloheximide (CHX) at the concentration of 0.5 μM for 24 hours.

Supplementary Table S1 Supplementary Table S1 shows primer sequences.

Supplementary Table S2 Supplementary Table S2 shows antibodies for immunophenotyping

Supplementary Table S3 Supplementary Table S3 shows gene descriptions in myeloma cells, following treatment with panobinostat or RG2833 using whole transcriptomic profiles.
