# Supplementary material for: HDAC Inhibition Induces CD26 Expression on Multiple Myeloma Cells via the c-Myc/Sp1-mediated Promoter Activation
Source: Cancer Res Commun. 2024 Feb 9;4(2):349–64. doi: 10.1158/2767-9764.CRC-23-0215 (PMC10854391; doi:10.1158/2767-9764.CRC-23-0215)
Supplement: Supplementary Methods S1 — include details of protocols for immunophenotyping (Supplementary Table S2), quantitation and qualification of mRNA levels, immunohistochemistry, immunoblotting, enzyme-linked immunosorbent assay (ELISA), and ChIP-qPCR. [file crc-23-0215-s01.docx]

**Supplementary Methods S1**

***Immunophenotyping***

Expression of cell surface proteins: CD26, CD38, BCMA/TNFRSF17, SLAMF7/CS1, CD55 and CD59 on five myeloma cell lines: KMS11, 26, 27, 28, and RPMI8226, and CD26 on immune effector cells including NK cells was determined by flow cytometry using specifically conjugated mAbs and isotype controls, as shown in Supplementary Materials. Flow cytometry analysis was performed on an EPICS XL-MCL^TM^ (Beckman Coulter, Brea, CA, USA) for single stain and CytoFLEX (Beckman Coulter) for double stain. Sorting procedure was performed using MoFlo (Beckman Coulter). The data were analyzed using FlowJo software, version. 10.9.2 (Tree Star, Ashland, OR, USA).

***Antibodies***

For details, see Supplementary Table S3.

***Quantitation and qualification of mRNA levels***

Total RNAs were extracted from five myeloma cell lines: KMS11, 26, 27, 28, and RPMI8226, suspended in trizol using chroloform and 2-propanol according to the manufacturer's instructions. The quality and integrity of each RNA was verified by a Bioanalyzer 2100 (Agilent Technologies, Santa Clara, CA, USA). Complementary DNA (cDNA) was synthesized from 1 µg of total RNA and reverse transcription‑quantitative polymerase chain reaction (RT-qPCR) was performed using specific primers with SYBR Premix Ex Taq II (Takara Bio, Shiga, Japan) on the Thermal Cycle Dice (Takara Bio) according to the standard PCR condition, consisting of the following: (quantification) 95˚C for 3 min for denaturation; 95˚C for 30 sec for annealing; and 62˚C for 40 sec for extension for 40 cycles and (qualification) 95˚C for 3 min for denaturation; 95˚C for 15 sec for annealing; and 60˚C for 60 sec for extension for 40 cycles . The primer sequences used in the experiments are described in Supplementary Table S1. Primers specific for β-actin were used as a control. The threshold cycle for each sample was selected from the linear range and converted to a starting quantity by interpolation from a standard curve generated on the same plate for each set of primers. The CD26 mRNA levels were normalized for each well to the GAPDH mRNA levels using the 2-ΔΔCq method (Livak *et al*. Methods, 2001).

***Immunohistochemistry***

In advance, bone marrow biopsy samples were fixed in 10% neutral-buffered formalin and embedded in paraffin. After prepared sections were deparaffinized and hydrated, the sections were washed, subjected to antigen retrieval for CD26 staining by autoclaving at 120℃ for 10 minutes in 0.01 M sodium citrate (pH6.0) and exposed to 3% H_2_O_2_ for 10 minutes to inactivate endogenous peroxidase. Subsequently, paraffin slides of viable myeloma cell lines (40,000 cells / slides) were incubated with methanol and 2.5% rabbit serum for 10 minutes, and then, tissue sections were incubated with goat anti-human CD26 polyclonal antibody (R&D systems, Minneapolis, MN, USA) at room temperature for 2 hours as a primary antibody, followed by the incubation with polyclonal rabbit anti-goat IgG1 antibody (Sigma Aldrich, St. Louis, MO, USA) as a secondary antibody for 30 min. Finally, the reaction was visualized with 3,3’-diaminobezidine (DAB) (Dojindo, Laboratories, Kumamoto, Japan) and the tissue sections were counterstained for nucleus with hematoxylin. The microscope by Zeiss (Jena, Germany) was used to observe their immunostaining and the images were processed using Nanozoomer-XR (Hamamatsu photonics, Hamamatsu, Japan).

***Immunoblotting***

Myeloma cell lines were collected and lysed in ice-cold lysis buffer, supplemented with 1 mM phenylmethylsulfonyl fluoride and proteasome inhibitor cocktail solution (Sigma Aldrich). The cell lysates were separated by sodium dodecyl sulfate polyacrylamide gel electrophoresis (SDS-PAGE) on a 7.5% polyacrylamide gel and transferred to PVDF membranes (Millipore, Billerica, MA, USA). The membranes were blocked with 5% skim-milk in Tris-buffered saline with 0.01% Tween for 2 hour at room temperature and incubated for 24 hours at 4℃ with each primary antibody as was shown below. After wash, the secondary horseradish peroxidase-conjugated antibody was added. The membranes were incubated with SuperSignal Atto West for 5 min (ThermoFisher Scientific, Waltham, MA, USA): and was visualized iBrightFL1000 (ThermoFisher Scientific). Rabbit anti-human c-Myc and c-Myc323Ac (Cell Signaling Technology, Danvers, MA, USA) was used as a primary antibody. Mouse anti-human β-actin was used as a benchmarker for total proteins. Horseradish peroxidase-conjugated goat anti-rabbit IgG-HRP (ImPRESS, Burlingame, CA, USA) and anti-mouse IgG-HRP (ImPRESS) were used as a secondary antibody.

***Enzyme-linked immunosorbent assay (ELISA)***

DPPⅣ levels in the supernatants from myeloma cell culture, treated with one of five HDACi (panoinostat, entinostat, romidepsin, ricolinostat, RG2833) or control IgG_1_ for 48 hours were assayed by human DPP4/Dipeptydil peptidase 4 ELISA kit (AB clonal, Woburn, MA, USA) according to the manufacturer’s instructions. Briefly, each standard and test samples were incubated in plates coated with DPPⅣ as a capture antibody. After the incubation for 2 hours at 37℃, biotin-conjugated anti-human DPPⅣ antibody was added and incubated for 1 hour at room temperature. Subsequently streptavidin-HRP was added, incubated for 0.5 hour, followed by the addition of substrate solution and incubation for 20 minutes at 37℃ under dark condition. Finally, the OD value of DPPⅣ in each sample was determined at 450 nm with GloMax-Muluti Detection System (Promega, Madison, WI, USA) and its DPPⅣ concentration was calculated using the standard curves.

***ChIP-qPCR***

ChIP assay was performed using truChIP chromatin shearing kit (Covaris, Woburn, MA, USA), SimpleChIP Plus Sonication Chromatin IP kit (Cell Signaling Technology, Danvers, MA, USA) according to the manufacturer’s instructions. Briefly, three myeloma cell lines; KMS11, 27, and RPMI8226 (3.0 x10^6^ cells/well for histone proteins or 1.0 x10^7^ cells/well for transcription factors) were harvested and incubated for 48 hours in the presence or absence of panobinostat or RG2833 and then fixed with 1% formaldehyde at 37°at RT for 10 min. Subsequently, cells were sonicated to obtain chromatin suspensions by AFA-Focused-Ultrasonicator (Covaris) under the determined condition; Intensity 200, Burst 100, 12 min. Thereafter, supernatants of diluted chromatin samples were mixed with the indicated antibodies and incubated at 4°for 4 hours to overnight, followed by the mixture with protein G magnetic beads at 4 ° for 2 hours. Beads were centrifuged. The 2% of sheared chromatin was kept aside to be used as an INPUT sample. DNA fragments in each IP samples, bound to the beads were purified using magnetic separation rack by repeated washing for 3 times with low salt-containing 1x ChIP buffer plus wash for 1 time with high salt containing buffer. After the completion of de-crosslink procedure in chromatin in IP samples were eluted with 1x DNA isolation buffer containing proteinase K and then, the crosslink was reversed by the incubation at 65 ° at 1200 rpm for 30 min, followed by the incubation at 65° for 2 hours with the addition of 5M NaCl plus RNase. Purified DNA samples were subjected to qPCR using primers as described in Supplementary Table S1 under the following PCR condition: initial denaturation at 95° for 3 min, followed by 40 cycles of denature at 95°for 15 sec plus annealing and extension at 60°for 1min. Recovery of ChIP’s DNA was calculated as a percentage of IP/INPUT. For ChIP-re-ChIP, pulled chromatin was eluted in 1% SDS buffer and diluted 10 times with elution buffer and repeated re-ChIP with the indicated antibody. After the completion of de-crosslink procedure, purified DNA samples were also subjected to qPCR.
