# Supplementary material for: HDAC Inhibition Induces CD26 Expression on Multiple Myeloma Cells via the c-Myc/Sp1-mediated Promoter Activation
Source: Cancer Res Commun. 2024 Feb 9;4(2):349–64. doi: 10.1158/2767-9764.CRC-23-0215 (PMC10854391; doi:10.1158/2767-9764.CRC-23-0215)
Supplement: Supplementary Figure S1 — shows effects of bortezomib or melphalan on CD26 expression on myeloma cells. [file crc-23-0215-s02.pptx]

## Slide 1
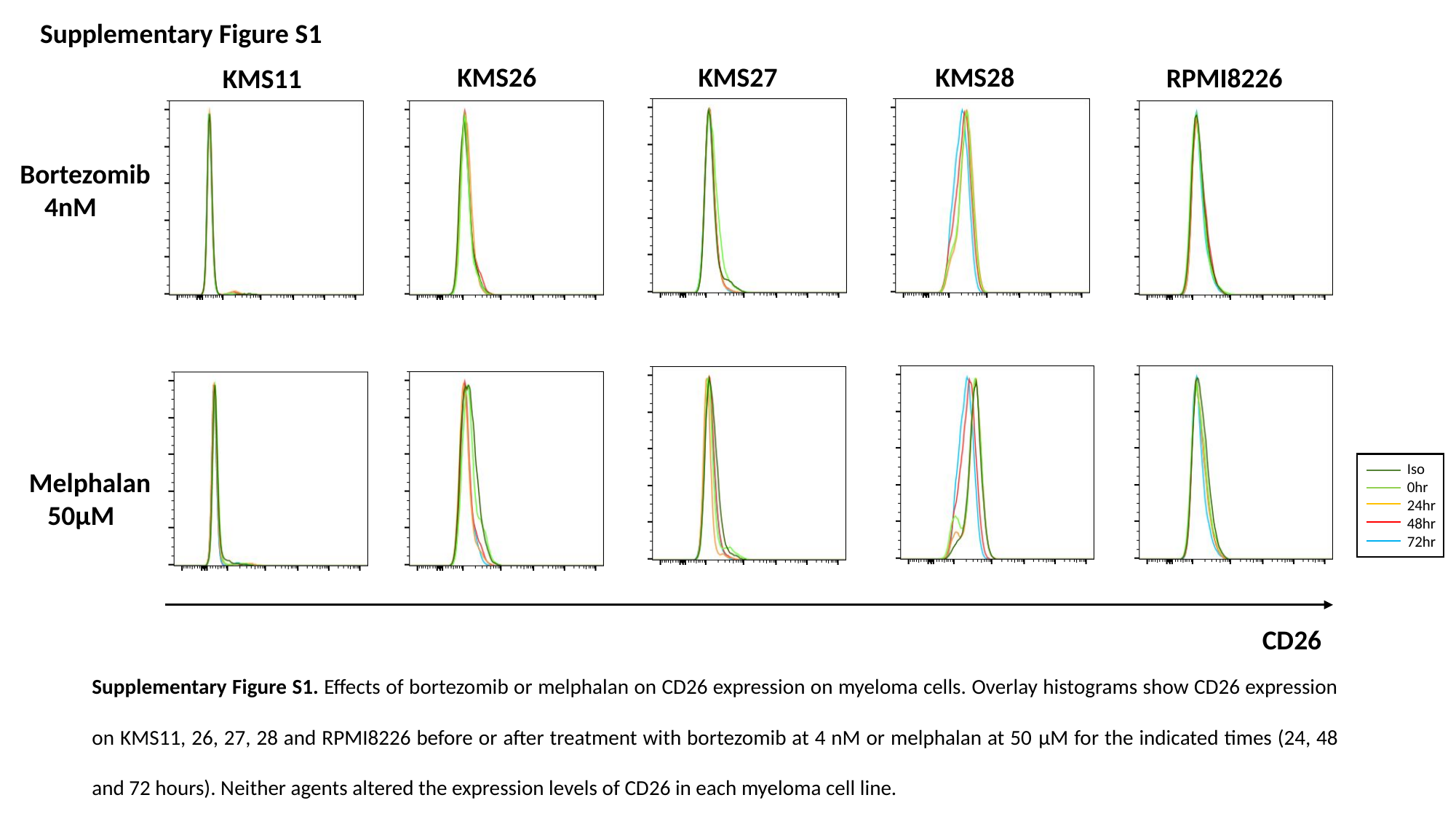

Supplementary Figure S1
KMS26
KMS27
KMS28
RPMI8226
KMS11
Bortezomib
 4nM
Iso
0hr
24hr
48hr
72hr
Melphalan
 50μM
CD26
Supplementary Figure S1. Effects of bortezomib or melphalan on CD26 expression on myeloma cells. Overlay histograms show CD26 expression on KMS11, 26, 27, 28 and RPMI8226 before or after treatment with bortezomib at 4 nM or melphalan at 50 μM for the indicated times (24, 48 and 72 hours). Neither agents altered the expression levels of CD26 in each myeloma cell line.
