# Supplementary material for: HDAC Inhibition Induces CD26 Expression on Multiple Myeloma Cells via the c-Myc/Sp1-mediated Promoter Activation
Source: Cancer Res Commun. 2024 Feb 9;4(2):349–64. doi: 10.1158/2767-9764.CRC-23-0215 (PMC10854391; doi:10.1158/2767-9764.CRC-23-0215)
Supplement: Supplementary Figure S2 — shows viability of KMS27 and KMS28, untreated or treated with panobinostat, RG2833 and entinostat at the indicated doses for 48 hours by MTT assay. [file crc-23-0215-s03.pptx]

## Slide 1
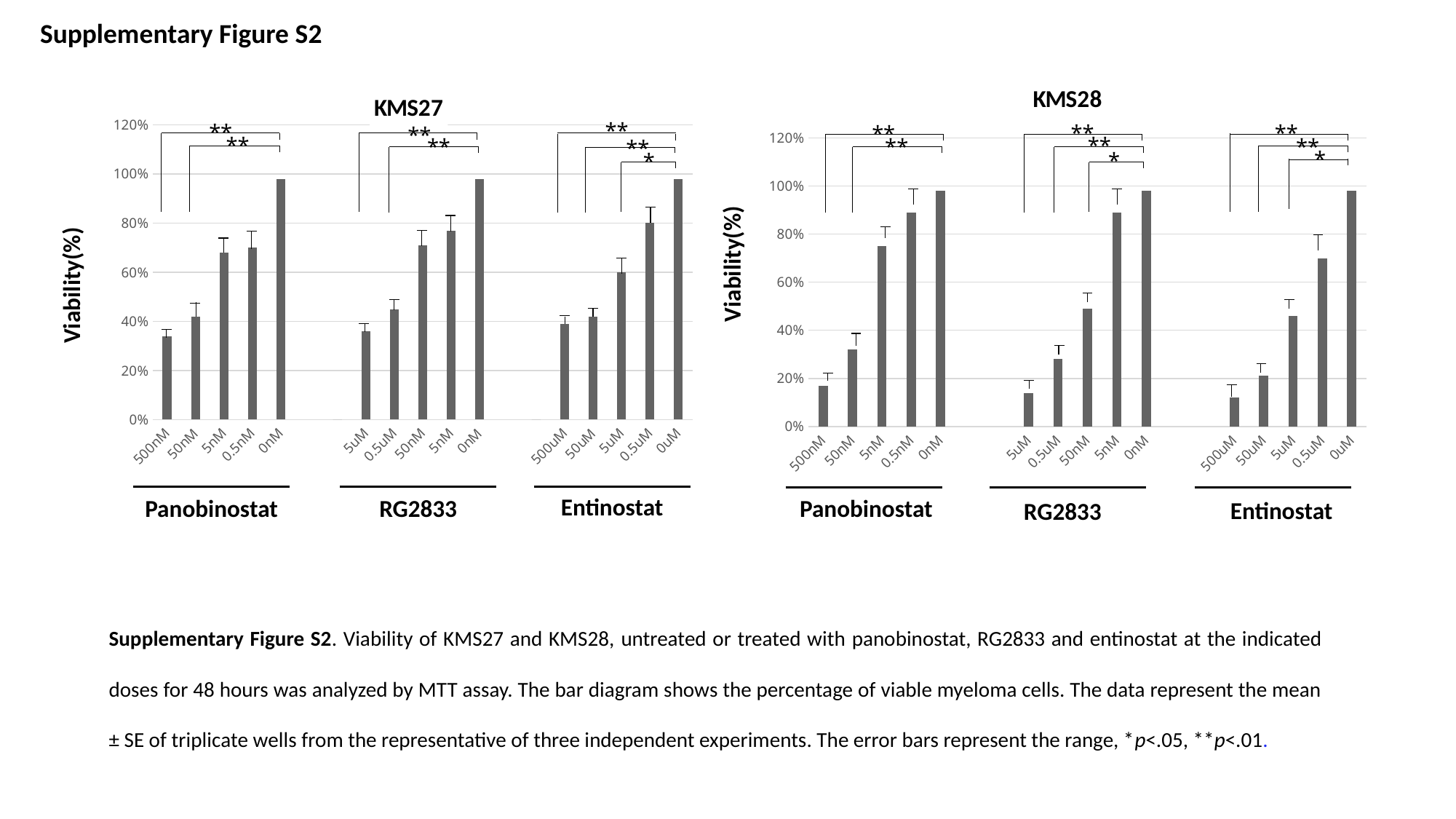

Supplementary Figure S2
### Chart: KMS28
| Category | Viability(%) |
|---|---|
| 500nM | 0.17 |
| 50nM | 0.32 |
| 5nM | 0.75 |
| 0.5nM | 0.89 |
| 0nM | 0.98 |
| | None |
| | None |
| 5uM | 0.14 |
| 0.5uM | 0.28 |
| 50nM | 0.49 |
| 5nM | 0.89 |
| 0nM | 0.98 |
| | None |
| | None |
| 500uM | 0.12 |
| 50uM | 0.21 |
| 5uM | 0.46 |
| 0.5uM | 0.7 |
| 0uM | 0.98 |
### Chart: KMS27
| Category | Viability |
|---|---|
| 500nM | 0.34 |
| 50nM | 0.42 |
| 5nM | 0.68 |
| 0.5nM | 0.7 |
| 0nM | 0.98 |
| | None |
| | 0.0 |
| 5uM | 0.36 |
| 0.5uM | 0.45 |
| 50nM | 0.71 |
| 5nM | 0.77 |
| 0nM | 0.98 |
| | None |
| | 0.0 |
| 500uM | 0.39 |
| 50uM | 0.42 |
| 5uM | 0.6 |
| 0.5uM | 0.8 |
| 0uM | 0.98 |**
**
**
**
**
**
**
**
**
**
**
**
*
*
*
Viability(%)
Viability(%)
Entinostat
Panobinostat
Panobinostat
RG2833
Entinostat
RG2833
Supplementary Figure S2. Viability of KMS27 and KMS28, untreated or treated with panobinostat, RG2833 and entinostat at the indicated doses for 48 hours was analyzed by MTT assay. The bar diagram shows the percentage of viable myeloma cells. The data represent the mean ± SE of triplicate wells from the representative of three independent experiments. The error bars represent the range, *p<.05, **p<.01.
