# Supplementary material for: HDAC Inhibition Induces CD26 Expression on Multiple Myeloma Cells via the c-Myc/Sp1-mediated Promoter Activation
Source: Cancer Res Commun. 2024 Feb 9;4(2):349–64. doi: 10.1158/2767-9764.CRC-23-0215 (PMC10854391; doi:10.1158/2767-9764.CRC-23-0215)
Supplement: Supplementary Figure S3 — shows effects of HDAC inhibition on CD38 expression on myeloma cells. [file crc-23-0215-s04.pptx]

## Slide 1
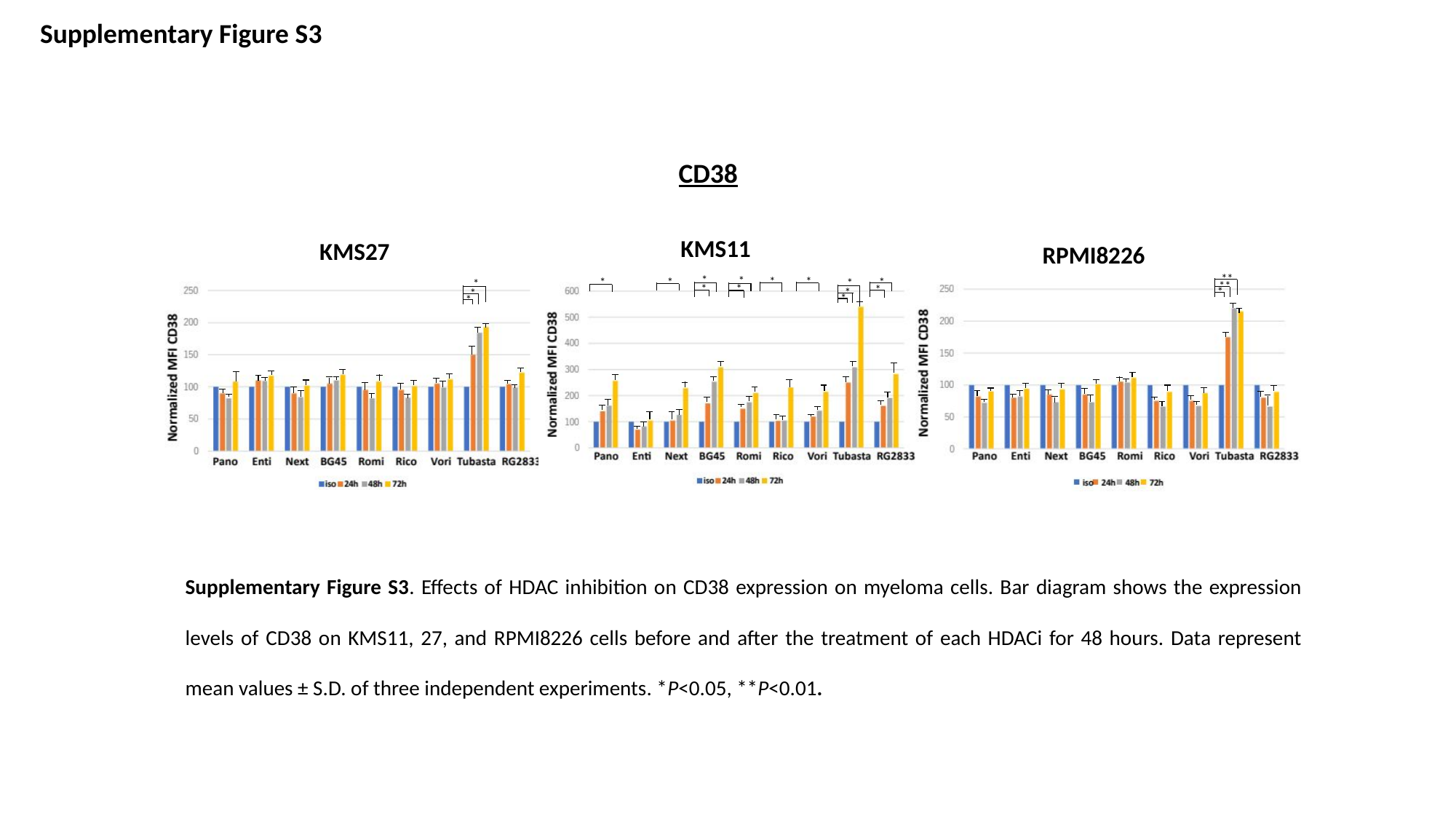

Supplementary Figure S3
CD38
KMS11
KMS27
RPMI8226
**
*
*
*
*
*
*
*
*
*
**
*
*
*
*
*
*
*
*
Supplementary Figure S3. Effects of HDAC inhibition on CD38 expression on myeloma cells. Bar diagram shows the expression levels of CD38 on KMS11, 27, and RPMI8226 cells before and after the treatment of each HDACi for 48 hours. Data represent mean values ± S.D. of three independent experiments. *P<0.05, **P<0.01.
