# Supplementary material for: HDAC Inhibition Induces CD26 Expression on Multiple Myeloma Cells via the c-Myc/Sp1-mediated Promoter Activation
Source: Cancer Res Commun. 2024 Feb 9;4(2):349–64. doi: 10.1158/2767-9764.CRC-23-0215 (PMC10854391; doi:10.1158/2767-9764.CRC-23-0215)
Supplement: Supplementary Figure S4 — shows effects of HDAC inhibition on BCMA expression on myeloma cells. [file crc-23-0215-s05.pptx]

## Slide 1
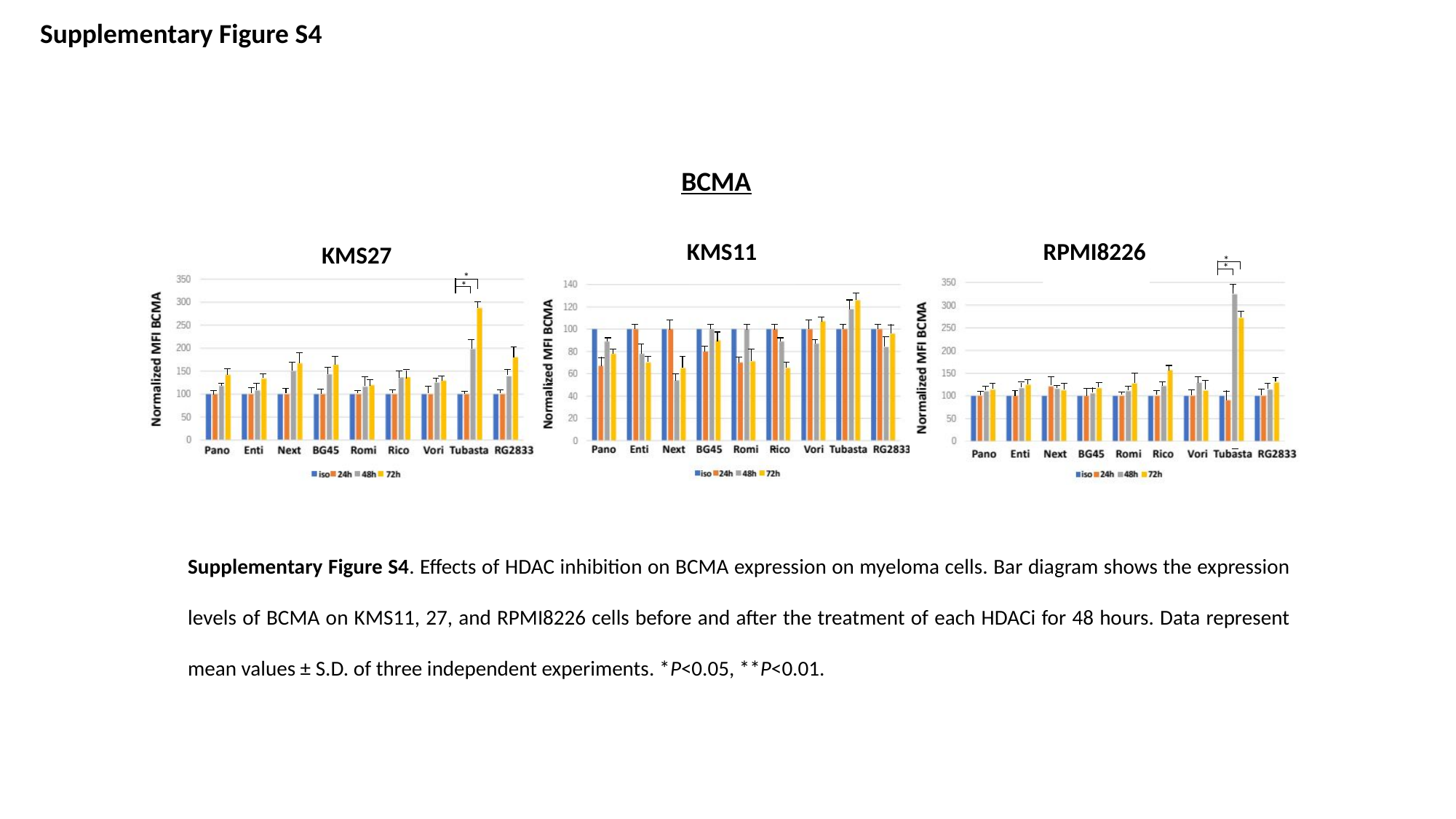

Supplementary Figure S4
BCMA
KMS11
RPMI8226
KMS27
*
*
*
*
Supplementary Figure S4. Effects of HDAC inhibition on BCMA expression on myeloma cells. Bar diagram shows the expression levels of BCMA on KMS11, 27, and RPMI8226 cells before and after the treatment of each HDACi for 48 hours. Data represent mean values ± S.D. of three independent experiments. *P<0.05, **P<0.01.
