# Supplementary material for: HDAC Inhibition Induces CD26 Expression on Multiple Myeloma Cells via the c-Myc/Sp1-mediated Promoter Activation
Source: Cancer Res Commun. 2024 Feb 9;4(2):349–64. doi: 10.1158/2767-9764.CRC-23-0215 (PMC10854391; doi:10.1158/2767-9764.CRC-23-0215)
Supplement: Supplementary Figure S5 — shows effects of HDAC inhibition on SLAMF7/CS1 expression on myeloma cells. [file crc-23-0215-s06.pptx]

## Slide 1
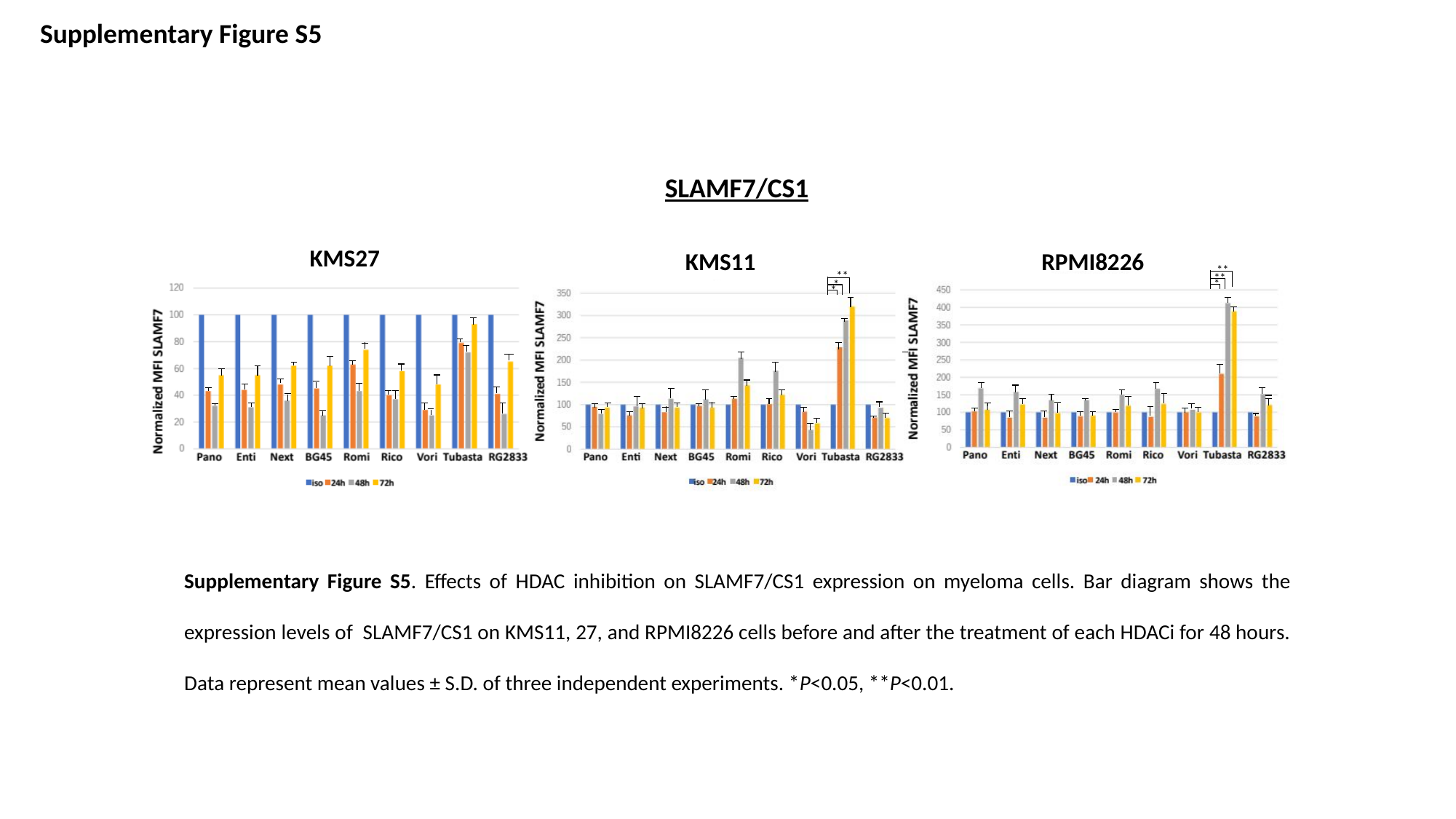

Supplementary Figure S5
SLAMF7/CS1
KMS27
KMS11
RPMI8226
**
**
**
*
*
*
Supplementary Figure S5. Effects of HDAC inhibition on SLAMF7/CS1 expression on myeloma cells. Bar diagram shows the expression levels of SLAMF7/CS1 on KMS11, 27, and RPMI8226 cells before and after the treatment of each HDACi for 48 hours. Data represent mean values ± S.D. of three independent experiments. *P<0.05, **P<0.01.
