# Supplementary material for: HDAC Inhibition Induces CD26 Expression on Multiple Myeloma Cells via the c-Myc/Sp1-mediated Promoter Activation
Source: Cancer Res Commun. 2024 Feb 9;4(2):349–64. doi: 10.1158/2767-9764.CRC-23-0215 (PMC10854391; doi:10.1158/2767-9764.CRC-23-0215)
Supplement: Supplementary Figure S6 — shows immunoblotting for c-Myc in KMS11, 27 and RPMI82226, treated with panobinostat or RG2833 in the presence or absence of cycloheximide (CHX) at the concentration of 0.5 μM for 24 hours. [file crc-23-0215-s07.pptx]

## Slide 1
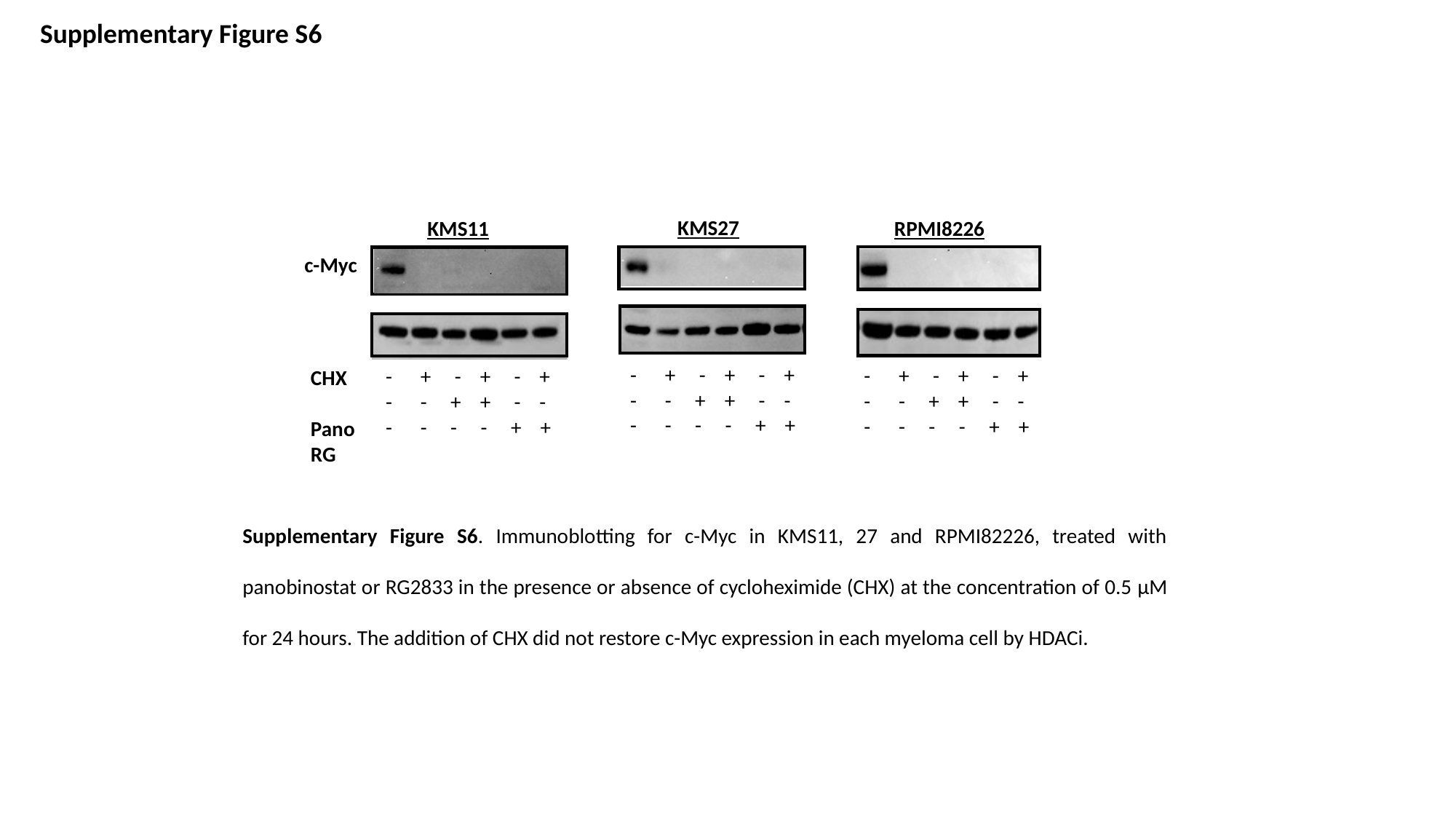

Supplementary Figure S6
KMS27
KMS11
RPMI8226
c-Myc
 - + - + - +
 - - + + - -
 - - - - + +
 - + - + - +
 - - + + - -
 - - - - + +
 - + - + - +
 - - + + - -
 - - - - + +
CHX
Pano
RG
Supplementary Figure S6. Immunoblotting for c-Myc in KMS11, 27 and RPMI82226, treated with panobinostat or RG2833 in the presence or absence of cycloheximide (CHX) at the concentration of 0.5 μM for 24 hours. The addition of CHX did not restore c-Myc expression in each myeloma cell by HDACi.
