# Supplementary material for: HDAC Inhibition Induces CD26 Expression on Multiple Myeloma Cells via the c-Myc/Sp1-mediated Promoter Activation
Source: Cancer Res Commun. 2024 Feb 9;4(2):349–64. doi: 10.1158/2767-9764.CRC-23-0215 (PMC10854391; doi:10.1158/2767-9764.CRC-23-0215)
Supplement: Supplementary Table S1 — shows primer sequences. [file crc-23-0215-s08.pptx]

## Slide 1
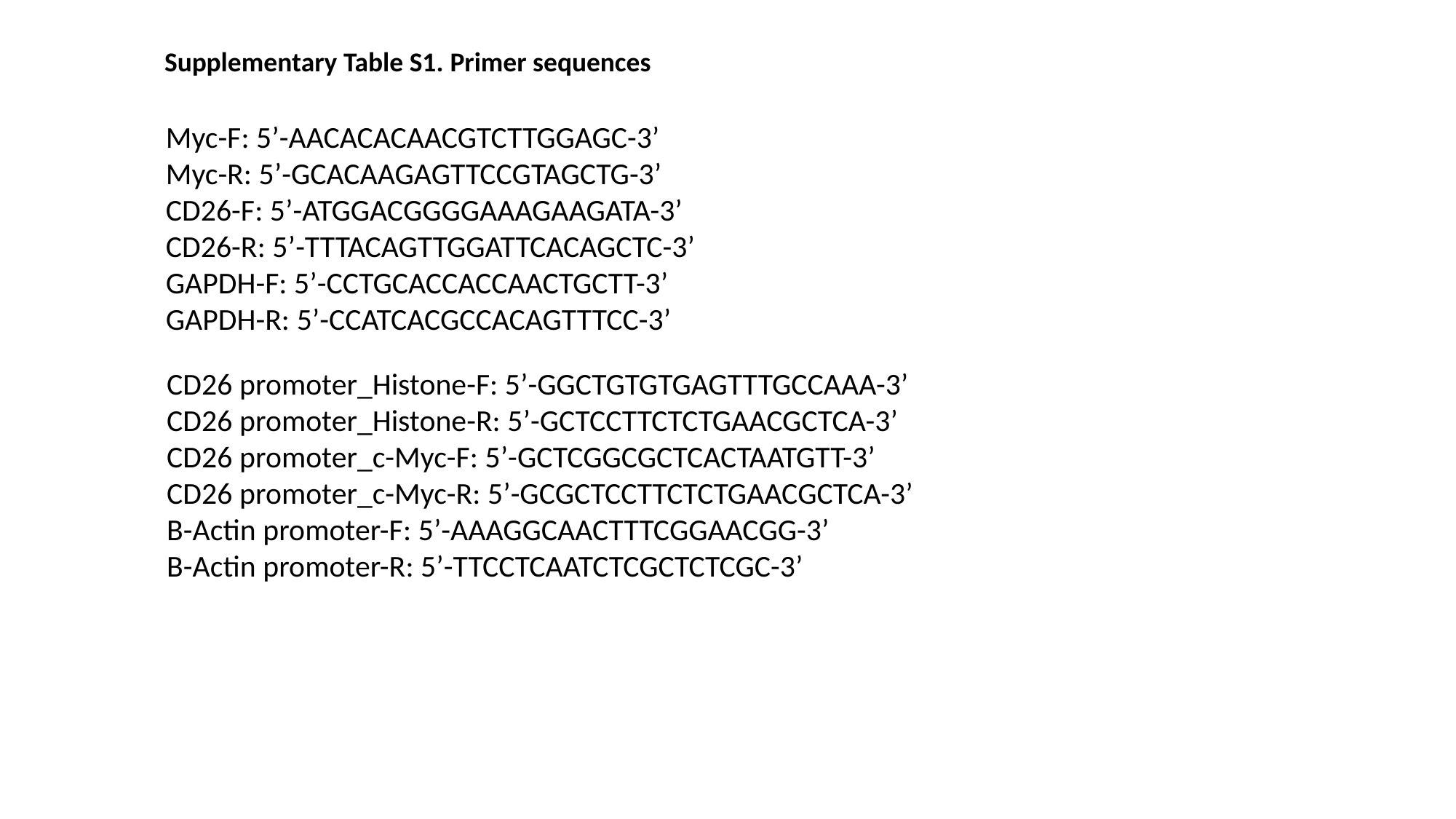

Supplementary Table S1. Primer sequences
Myc-F: 5’-AACACACAACGTCTTGGAGC-3’
Myc-R: 5’-GCACAAGAGTTCCGTAGCTG-3’
CD26-F: 5’-ATGGACGGGGAAAGAAGATA-3’
CD26-R: 5’-TTTACAGTTGGATTCACAGCTC-3’
GAPDH-F: 5’-CCTGCACCACCAACTGCTT-3’
GAPDH-R: 5’-CCATCACGCCACAGTTTCC-3’
CD26 promoter_Histone-F: 5’-GGCTGTGTGAGTTTGCCAAA-3’
CD26 promoter_Histone-R: 5’-GCTCCTTCTCTGAACGCTCA-3’
CD26 promoter_c-Myc-F: 5’-GCTCGGCGCTCACTAATGTT-3’
CD26 promoter_c-Myc-R: 5’-GCGCTCCTTCTCTGAACGCTCA-3’
B-Actin promoter-F: 5’-AAAGGCAACTTTCGGAACGG-3’
B-Actin promoter-R: 5’-TTCCTCAATCTCGCTCTCGC-3’
