# Supplementary material for: HDAC Inhibition Induces CD26 Expression on Multiple Myeloma Cells via the c-Myc/Sp1-mediated Promoter Activation
Source: Cancer Res Commun. 2024 Feb 9;4(2):349–64. doi: 10.1158/2767-9764.CRC-23-0215 (PMC10854391; doi:10.1158/2767-9764.CRC-23-0215)
Supplement: Supplementary Table S2 — shows antibodies for immunophenotyping [file crc-23-0215-s09.pptx]

## Slide 1
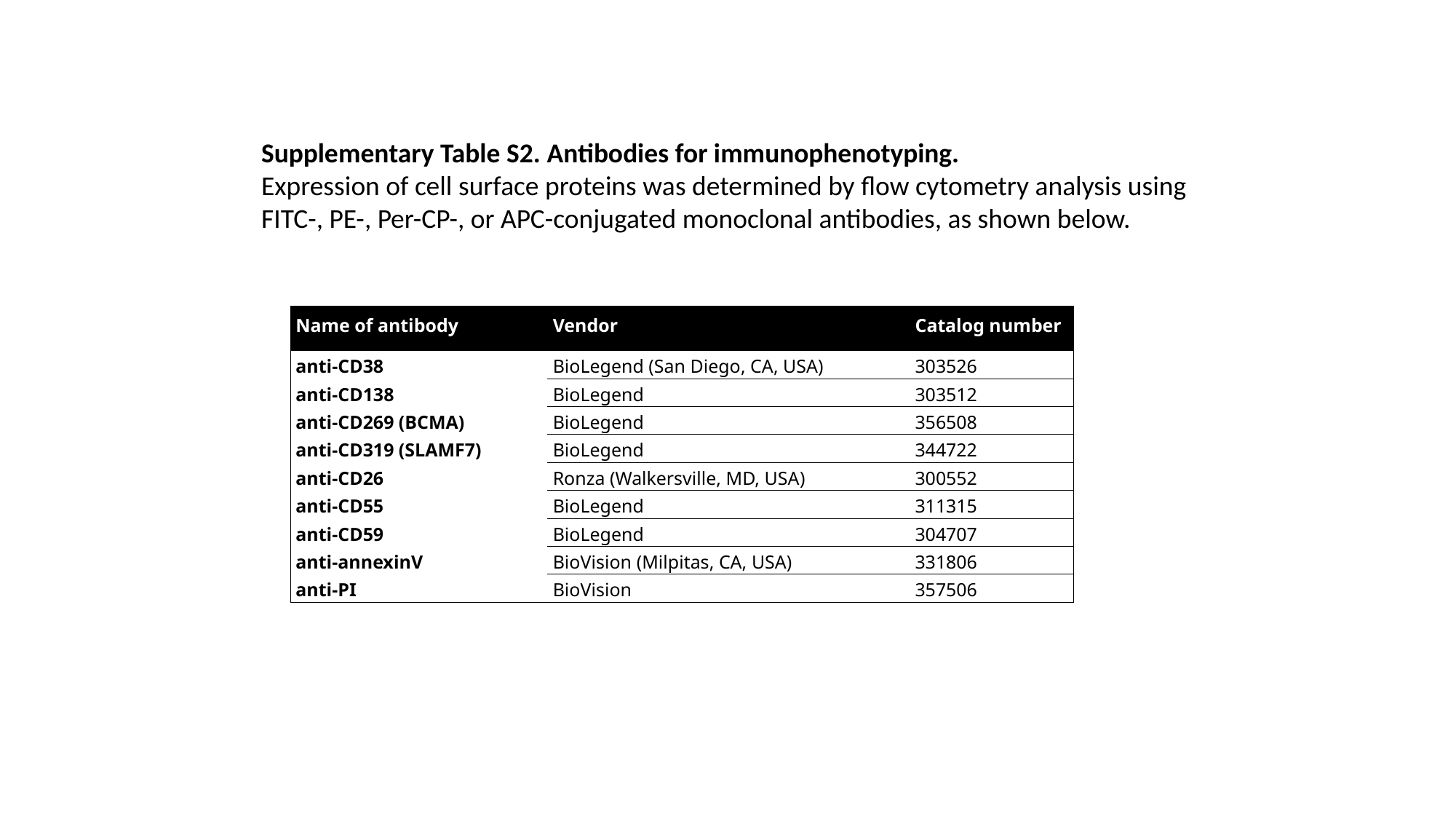

Supplementary Table S2. Antibodies for immunophenotyping.
Expression of cell surface proteins was determined by flow cytometry analysis using
FITC-, PE-, Per-CP-, or APC-conjugated monoclonal antibodies, as shown below.
| Name of antibody | Vendor | Catalog number |
| --- | --- | --- |
| anti-CD38 | BioLegend (San Diego, CA, USA) | 303526 |
| anti-CD138 | BioLegend | 303512 |
| anti-CD269 (BCMA) | BioLegend | 356508 |
| anti-CD319 (SLAMF7) | BioLegend | 344722 |
| anti-CD26 | Ronza (Walkersville, MD, USA) | 300552 |
| anti-CD55 | BioLegend | 311315 |
| anti-CD59 | BioLegend | 304707 |
| anti-annexinV | BioVision (Milpitas, CA, USA) | 331806 |
| anti-PI | BioVision | 357506 |
