# Supplementary material for: HDAC Inhibition Induces CD26 Expression on Multiple Myeloma Cells via the c-Myc/Sp1-mediated Promoter Activation
Source: Cancer Res Commun. 2024 Feb 9;4(2):349–64. doi: 10.1158/2767-9764.CRC-23-0215 (PMC10854391; doi:10.1158/2767-9764.CRC-23-0215)
Supplement: Supplementary Table S3 — shows gene descriptions in myeloma cells, following treatment with panobinostat or RG2833 using whole transcriptomic profiles. [file crc-23-0215-s10.pptx]

## Slide 1
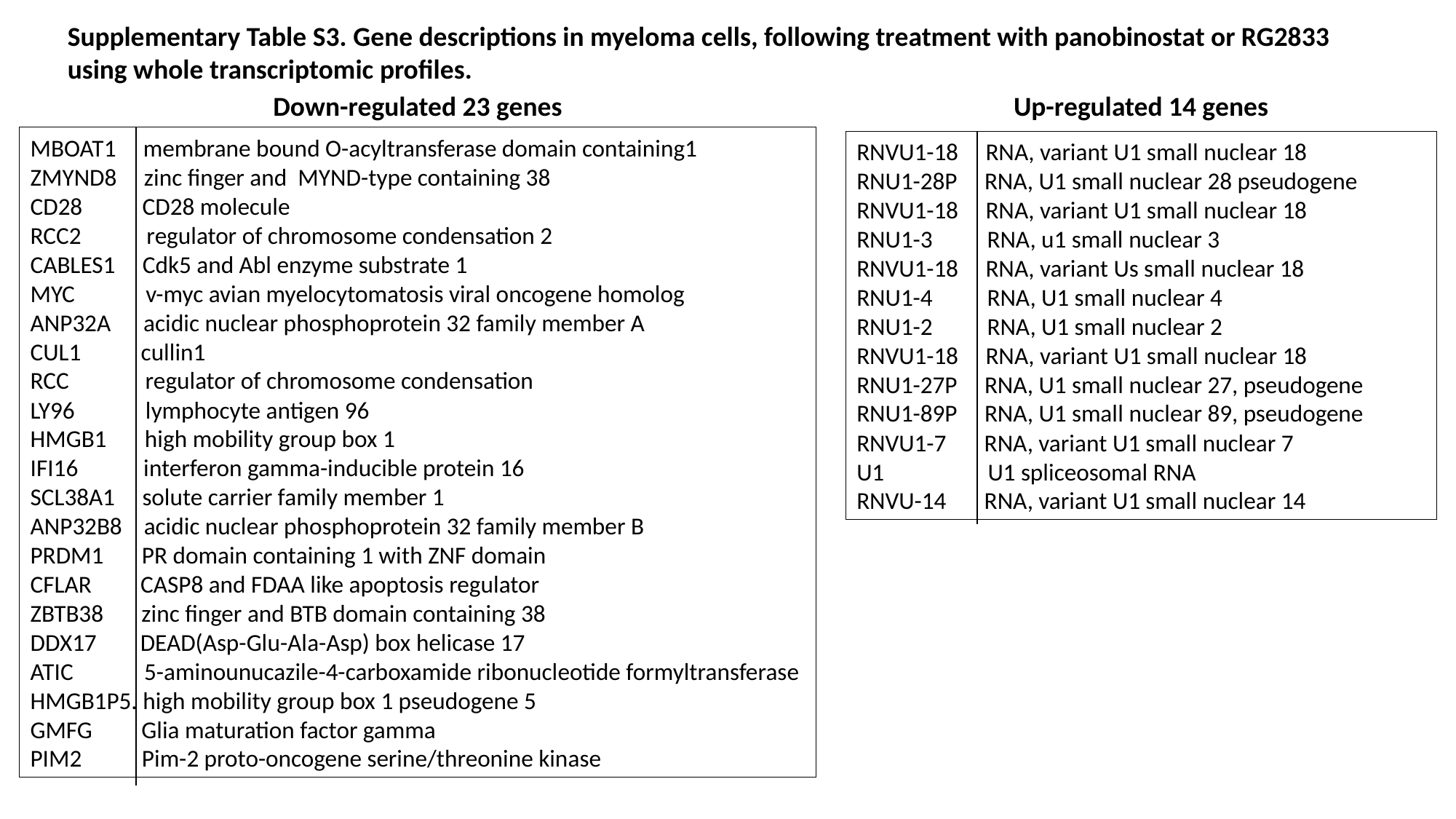

Supplementary Table S3. Gene descriptions in myeloma cells, following treatment with panobinostat or RG2833 using whole transcriptomic profiles.
Down-regulated 23 genes
Up-regulated 14 genes
MBOAT1 membrane bound O-acyltransferase domain containing1
ZMYND8 zinc finger and MYND-type containing 38
CD28 CD28 molecule
RCC2 regulator of chromosome condensation 2
CABLES1 Cdk5 and Abl enzyme substrate 1
MYC v-myc avian myelocytomatosis viral oncogene homolog
ANP32A acidic nuclear phosphoprotein 32 family member A
CUL1 cullin1
RCC regulator of chromosome condensation
LY96 lymphocyte antigen 96
HMGB1 high mobility group box 1
IFI16 interferon gamma-inducible protein 16
SCL38A1 solute carrier family member 1
ANP32B8 acidic nuclear phosphoprotein 32 family member B
PRDM1 PR domain containing 1 with ZNF domain
CFLAR CASP8 and FDAA like apoptosis regulator
ZBTB38 zinc finger and BTB domain containing 38
DDX17 DEAD(Asp-Glu-Ala-Asp) box helicase 17
ATIC 5-aminounucazile-4-carboxamide ribonucleotide formyltransferase
HMGB1P5. high mobility group box 1 pseudogene 5
GMFG Glia maturation factor gamma
PIM2 Pim-2 proto-oncogene serine/threonine kinase
RNVU1-18 RNA, variant U1 small nuclear 18
RNU1-28P RNA, U1 small nuclear 28 pseudogene
RNVU1-18 RNA, variant U1 small nuclear 18
RNU1-3 RNA, u1 small nuclear 3
RNVU1-18 RNA, variant Us small nuclear 18
RNU1-4 RNA, U1 small nuclear 4
RNU1-2 RNA, U1 small nuclear 2
RNVU1-18 RNA, variant U1 small nuclear 18
RNU1-27P RNA, U1 small nuclear 27, pseudogene
RNU1-89P RNA, U1 small nuclear 89, pseudogene
RNVU1-7 RNA, variant U1 small nuclear 7
U1 U1 spliceosomal RNA
RNVU-14 RNA, variant U1 small nuclear 14
